# Supplementary material for: Social support receipt as a predictor of mortality: A cohort study in rural South Africa
Source: PLOS Glob Public Health. 2024 Sep 9;4(9):e0003683. doi: 10.1371/journal.pgph.0003683 (PMC11383236; doi:10.1371/journal.pgph.0003683)
Supplement: S1 Table — (PDF) [file pgph.0003683.s001.pdf]

S1 Table: Cox Proportional Hazard Models, Full - No Interaction

	Informational		Emotional		Financial		Physical	
	Hazard Ratio	Confidence Interval	Hazard Ratio	Confidence Interval	Hazard Ratio	Confidence Interval	Hazard Ratio	Confidence Interval
Social support	1.09	[0.99,1.18]	1.08	[1.00,1.18]	1.04	[0.95,1.13]	1.07	[0.98,1.16]
Sex (Male)	2.14***	[1.72,2.66]	2.05***	[1.65,2.54]	2.05***	[1.65,2.54]	2.06***	[1.65,2.56]
Never Married	2.07***	[1.37,3.13]	2.13***	[1.42,3.21]	2.14***	[1.42,3.23]	2.04***	[1.35,3.07]
Married/Partner	1	[1.00,1.00]	1	[1.00,1.00]	1	[1.00,1.00]	1	[1.00,1.00]
Separated/Deserted/Divorced	1.46**	[1.10,1.94]	1.45*	[1.09,1.92]	1.49**	[1.12,1.98]	1.46**	[1.10,1.94]
Widowed	1.35*	[1.07,1.70]	1.33*	[1.05,1.68]	1.35*	[1.07,1.71]	1.30*	[1.02,1.64]
Pension	1.13	[0.93,1.38]	1.15	[0.94,1.40]	1.15	[0.94,1.41]	1.12	[0.92,1.36]
Employed	0.7	[0.49,1.02]	0.69*	[0.48,1.00]	0.69*	[0.47,0.99]	0.73	[0.51,1.06]
Unemployed	1	[1.00,1.00]	1	[1.00,1.00]	1	[1.00,1.00]	1	[1.00,1.00]
Homemaker	0.97	[0.72,1.30]	1.04	[0.77,1.39]	1	[0.74,1.34]	1.09	[0.81,1.46]
40-49	1	[1.00,1.00]	1	[1.00,1.00]	1	[1.00,1.00]	1	[1.00,1.00]
50-59	2.31***	[1.47,3.62]	2.39***	[1.52,3.73]	2.33***	[1.49,3.64]	2.29***	[1.46,3.58]
60-69	2.68***	[1.68,4.28]	2.79***	[1.75,4.46]	2.72***	[1.70,4.35]	2.75***	[1.72,4.39]
70-79	3.53***	[2.17,5.74]	3.66***	[2.26,5.95]	3.59***	[2.20,5.85]	3.39***	[2.09,5.52]
80+	6.67***	[4.04,11.01]	6.79***	[4.12,11.20]	7.02***	[4.25,11.59]	5.83***	[3.52,9.67]
HIV Positive	1	[1.00,1.00]	1	[1.00,1.00]	1	[1.00,1.00]	1	[1.00,1.00]
HIV Negative	0.72**	[0.57,0.91]	0.72**	[0.57,0.92]	0.72**	[0.56,0.91]	0.69**	[0.54,0.88]
Missing HIV Data	0.87	[0.52,1.45]	0.81	[0.48,1.34]	0.84	[0.50,1.39]	0.87	[0.52,1.45]
Normal Anemia	1	[1.00,1.00]	1	[1.00,1.00]	1	[1.00,1.00]	1	[1.00,1.00]
Mild Anemia	1.19	[0.95,1.50]	1.17	[0.93,1.47]	1.2	[0.96,1.51]	1.21	[0.97,1.52]
Moderate Anemia	2.02***	[1.58,2.57]	1.96***	[1.54,2.50]	1.99***	[1.56,2.54]	1.93***	[1.52,2.46]
Severe Anemia	3.55***	[2.27,5.57]	3.66***	[2.33,5.74]	3.55***	[2.27,5.57]	3.30***	[2.10,5.17]
Intentional Refusal - Anemia	1.11	[0.47,2.61]	1.23	[0.52,2.93]	1.13	[0.48,2.65]	1.04	[0.44,2.46]
Processing Error - Anemia	1.55*	[1.02,2.37]	1.54*	[1.01,2.34]	1.58*	[1.03,2.40]	1.46	[0.96,2.24]
Hypertensive	1	[1.00,1.00]	1	[1.00,1.00]	1	[1.00,1.00]	1	[1.00,1.00]
Not Hypertensive	0.88	[0.72,1.07]	0.89	[0.73,1.08]	0.88	[0.72,1.08]	0.88	[0.72,1.07]
Intentional Refusal - Hypertension	1.21	[0.63,2.32]	1.27	[0.66,2.44]	1.23	[0.64,2.37]	1.36	[0.71,2.63]
Processing Error - Hypertension	1.65	[0.61,4.49]	1.85	[0.68,5.04]	1.7	[0.63,4.64]	1.83	[0.67,4.97]
Underweight	1.62**	[1.17,2.22]	1.55**	[1.12,2.14]	1.66**	[1.21,2.29]	1.31	[0.94,1.84]
Normal	1	[1.00,1.00]	1	[1.00,1.00]	1	[1.00,1.00]	1	[1.00,1.00]
Overweight	0.87	[0.68,1.11]	0.85	[0.66,1.09]	0.85	[0.67,1.09]	0.89	[0.70,1.14]

Obese	0.79	[0.60,1.04]	0.79	[0.60,1.03]	0.77	[0.59,1.02]	0.82	[0.62,1.07]
Miscellaneous Errors - BMI	3.00***	[2.28,3.93]	2.79***	[2.11,3.68]	3.00***	[2.29,3.94]	2.85**	[1.50,5.42]
No Diabetes/Not Fasting	0.63***	[0.50,0.81]	0.65***	[0.51,0.84]	0.63***	[0.49,0.80]	0.66***	[0.52,0.84]
Diabetic	1	[1.00,1.00]	1	[1.00,1.00]	1	[1.00,1.00]	1	[1.00,1.00]
Not Missing on Diabetes	1.45	[0.72,2.94]	1.51	[0.75,3.04]	1.45	[0.72,2.92]	1.43	[0.71,2.88]
Missing on Diabetes	1	[1.00,1.00]	1	[1.00,1.00]	1	[1.00,1.00]	1	[1.00,1.00]
No Formal Education	1	[1.00,1.00]	1	[1.00,1.00]	1	[1.00,1.00]	1	[1.00,1.00]
Some Primary Education (1-7 years)	1.01	[0.78,1.30]	0.9	[0.73,1.10]	0.87	[0.71,1.07]	0.98	[0.80,1.21]
Some Secondary Education (8-11 years)	1.02	[0.68,1.53]	0.9	[0.63,1.29]	0.85	[0.59,1.24]	1.01	[0.70,1.46]
Secondary Education or more (12+ years)	0.61	[0.34,1.12]	0.57	[0.32,1.01]	0.49*	[0.27,0.88]	0.64	[0.36,1.14]
Born in South Africa	1.2	[0.98,1.48]						
Literacy	0.75*	[0.58,0.95]						
PTSD			0.6	[0.33,1.10]				
Respondent depression			1.07**	[1.02,1.13]				
Father's education					1.06	[0.91,1.22]		
Lowest Wealth					1	[1.00,1.00]		
Low/Mid Wealth					0.93	[0.71,1.22]		
Medium Wealth					1.05	[0.80,1.37]		
Mid/High Wealth					1.01	[0.76,1.35]		
Highest Wealth					1.23	[0.91,1.65]		
Total cognitive score							0.98*	[0.96,1.00]
Missing on cognitive measure							0.52	[0.16,1.68]
Non-Frail							1	[1.00,1.00]
Pre-Frail							1.28*	[1.02,1.61]
Frail							1.98***	[1.33,2.96]
Unable to Score							1.11	[0.58,2.10]
ADL limitation							1.38*	[1.07,1.78]

N for all models is 4907, Exponentiated coefficients; 95% confidence intervals in brackets

* $p<0.05$, ** $p<0.01$, *** $p<0.001$
