# Supplementary material for: Social support receipt as a predictor of mortality: A cohort study in rural South Africa
Source: PLOS Glob Public Health. 2024 Sep 9;4(9):e0003683. doi: 10.1371/journal.pgph.0003683 (PMC11383236; doi:10.1371/journal.pgph.0003683)
Supplement: S2 Table — (PDF) [file pgph.0003683.s002.pdf]

S2 Table: Cox Proportional Hazard Models, Full - Sex Interaction

	Informational		Emotional		Financial		Physical	
	Hazard Ratio	Confidence Interval	Hazard Ratio	Confidence Interval	Hazard Ratio	Confidence Interval	Hazard Ratio	Confidence Interval
Female x Social support	1.13	[1.00,1.29]	1.1	[0.97,1.25]	1.1	[0.97,1.25]	1.06	[0.93,1.21]
Male x Social support	1.05	[0.94,1.18]	1.07	[0.96,1.19]	1	[0.89,1.12]	1.07	[0.96,1.20]
Sex (Male)	2.14***	[1.72,2.66]	2.05***	[1.65,2.54]	2.05***	[1.65,2.54]	2.06***	[1.65,2.56]
Never Married	2.06***	[1.37,3.11]	2.13***	[1.41,3.20]	2.15***	[1.42,3.23]	2.04***	[1.35,3.08]
Married/Partner	1	[1.00,1.00]	1	[1.00,1.00]	1	[1.00,1.00]	1	[1.00,1.00]
Separated/Deserted/Divorced	1.45**	[1.09,1.93]	1.44*	[1.09,1.92]	1.49**	[1.12,1.98]	1.46**	[1.10,1.94]
Widowed	1.34*	[1.06,1.70]	1.33*	[1.05,1.68]	1.34*	[1.06,1.70]	1.30*	[1.02,1.64]
Pension	1.13	[0.93,1.38]	1.15	[0.94,1.40]	1.15	[0.94,1.40]	1.12	[0.92,1.37]
Employed	0.7	[0.48,1.02]	0.69*	[0.48,1.00]	0.69*	[0.47,0.99]	0.73	[0.51,1.07]
Unemployed	1	[1.00,1.00]	1	[1.00,1.00]	1	[1.00,1.00]	1	[1.00,1.00]
Homemaker	0.97	[0.72,1.30]	1.04	[0.77,1.39]	1	[0.74,1.34]	1.08	[0.81,1.46]
40-49	1	[1.00,1.00]	1	[1.00,1.00]	1	[1.00,1.00]	1	[1.00,1.00]
50-59	2.31***	[1.48,3.62]	2.39***	[1.52,3.73]	2.32***	[1.48,3.64]	2.29***	[1.46,3.58]
60-69	2.69***	[1.68,4.29]	2.80***	[1.75,4.47]	2.74***	[1.71,4.39]	2.75***	[1.72,4.38]
70-79	3.55***	[2.18,5.78]	3.67***	[2.26,5.96]	3.62***	[2.22,5.90]	3.39***	[2.08,5.51]
80+	6.70***	[4.06,11.08]	6.81***	[4.13,11.23]	7.05***	[4.27,11.65]	5.83***	[3.51,9.66]
HIV Positive	1	[1.00,1.00]	1	[1.00,1.00]	1	[1.00,1.00]	1	[1.00,1.00]
HIV Negative	0.72**	[0.57,0.91]	0.72**	[0.57,0.92]	0.72**	[0.56,0.91]	0.69**	[0.54,0.88]
Missing HIV Data	0.87	[0.53,1.45]	0.81	[0.48,1.34]	0.84	[0.50,1.39]	0.87	[0.52,1.45]
Normal Anemia	1	[1.00,1.00]	1	[1.00,1.00]	1	[1.00,1.00]	1	[1.00,1.00]
Mild Anemia	1.19	[0.95,1.50]	1.17	[0.93,1.47]	1.2	[0.96,1.50]	1.21	[0.97,1.52]
Moderate Anemia	2.01***	[1.58,2.56]	1.96***	[1.54,2.49]	1.98***	[1.56,2.53]	1.93***	[1.52,2.46]
Severe Anemia	3.59***	[2.29,5.63]	3.67***	[2.34,5.76]	3.62***	[2.30,5.68]	3.29***	[2.09,5.16]
Intentional Refusal - Anemia	1.1	[0.46,2.59]	1.23	[0.52,2.93]	1.11	[0.47,2.64]	1.04	[0.44,2.46]
Processing Error - Anemia	1.56*	[1.02,2.37]	1.54*	[1.01,2.34]	1.58*	[1.04,2.41]	1.46	[0.96,2.24]
Hypertensive	1	[1.00,1.00]	1	[1.00,1.00]	1	[1.00,1.00]	1	[1.00,1.00]
Not Hypertensive	0.88	[0.72,1.07]	0.89	[0.73,1.08]	0.88	[0.72,1.07]	0.88	[0.72,1.08]
Intentional Refusal - Hypertension	1.2	[0.63,2.30]	1.27	[0.66,2.44]	1.24	[0.64,2.39]	1.36	[0.71,2.63]
Processing Error - Hypertension	1.63	[0.60,4.44]	1.85	[0.68,5.02]	1.7	[0.62,4.62]	1.83	[0.67,4.98]
Underweight	1.62**	[1.18,2.24]	1.55**	[1.12,2.14]	1.67**	[1.21,2.30]	1.31	[0.93,1.84]

Normal	1	[1.00,1.00]	1	[1.00,1.00]	1	[1.00,1.00]	1	[1.00,1.00]
Overweight	0.87	[0.68,1.11]	0.85	[0.66,1.09]	0.86	[0.67,1.10]	0.89	[0.70,1.14]
Obese	0.79	[0.60,1.04]	0.79	[0.60,1.03]	0.77	[0.59,1.02]	0.82	[0.62,1.07]
Miscellaneous Errors - BMI	3.02***	[2.30,3.96]	2.79***	[2.11,3.68]	3.01***	[2.29,3.95]	2.85**	[1.50,5.43]
No Diabetes/Not Fasting	0.63***	[0.50,0.81]	0.65***	[0.51,0.84]	0.63***	[0.50,0.81]	0.66***	[0.52,0.84]
Diabetic	1	[1.00,1.00]	1	[1.00,1.00]	1	[1.00,1.00]	1	[1.00,1.00]
Not Missing on Diabetes	1.45	[0.71,2.94]	1.5	[0.74,3.04]	1.45	[0.72,2.92]	1.43	[0.71,2.88]
Missing on Diabetes	1	[1.00,1.00]	1	[1.00,1.00]	1	[1.00,1.00]	1	[1.00,1.00]
No Formal Education	1	[1.00,1.00]	1	[1.00,1.00]	1	[1.00,1.00]	1	[1.00,1.00]
Some Primary Education (1-7 years)	1.01	[0.78,1.30]	0.9	[0.74,1.10]	0.87	[0.71,1.07]	0.99	[0.80,1.22]
Some Secondary Education (8-11 years)	1.02	[0.68,1.54]	0.9	[0.63,1.29]	0.85	[0.59,1.24]	1.01	[0.70,1.46]
Secondary Education or more (12+ years)	0.62	[0.34,1.12]	0.57	[0.32,1.01]	0.49*	[0.27,0.88]	0.64	[0.36,1.14]
Born in South Africa	1.21	[0.98,1.48]						
Literacy	0.75*	[0.59,0.96]						
PTSD			0.6	[0.33,1.10]				
Respondent depression			1.07**	[1.02,1.13]				
Father's education					1.06	[0.91,1.22]		
Lowest Wealth					1	[1.00,1.00]		
Low/Mid Wealth					0.94	[0.72,1.23]		
Medium Wealth					1.05	[0.80,1.38]		
Mid/High Wealth					1.02	[0.77,1.36]		
Highest Wealth					1.23	[0.91,1.66]		
Total cognitive score							0.98*	[0.96,1.00]
Missing on cognitive measure							0.52	[0.16,1.68]
Non-Frail							1	[1.00,1.00]
Pre-Frail							1.28*	[1.02,1.61]
Frail							1.98***	[1.33,2.96]
Unable to Score							1.11	[0.58,2.10]
ADL limitation							1.37*	[1.06,1.77]

N for all models is 4907, Exponentiated coefficients; 95% confidence intervals in brackets

* p<0.05, ** p<0.01, *** p<0.001
