# Supplementary material for: Social support receipt as a predictor of mortality: A cohort study in rural South Africa
Source: PLOS Glob Public Health. 2024 Sep 9;4(9):e0003683. doi: 10.1371/journal.pgph.0003683 (PMC11383236; doi:10.1371/journal.pgph.0003683)
Supplement: S3 Table — (PDF) [file pgph.0003683.s003.pdf]

S3 Table: Cox Proportional Hazard Models, Full - Age Interaction

	Informational		Emotional		Financial		Physical	
	Hazard Ratio	Confidence Interval	Hazard Ratio	Confidence Interval	Hazard Ratio	Confidence Interval	Hazard Ratio	Confidence Interval
Social support x Under 60	1.15	[0.97,1.35]	1.11	[0.93,1.31]	0.95	[0.78,1.16]	1.1	[0.93,1.31]
Social support x Older/Equal to 60	1.06	[0.96,1.18]	1.08	[0.98,1.18]	1.06	[0.97,1.17]	1.06	[0.96,1.16]
Sex (Male)	2.14***	[1.72,2.66]	2.05***	[1.65,2.54]	2.04***	[1.65,2.54]	2.06***	[1.66,2.56]
Never Married	2.09***	[1.38,3.15]	2.13***	[1.42,3.21]	2.14***	[1.42,3.22]	2.05***	[1.36,3.10]
Married/Partner	1	[1.00,1.00]	1	[1.00,1.00]	1	[1.00,1.00]	1	[1.00,1.00]
Separated/Deserted/Divorced	1.46**	[1.10,1.94]	1.45*	[1.09,1.92]	1.49**	[1.12,1.98]	1.46**	[1.10,1.94]
Widowed	1.34*	[1.06,1.70]	1.33*	[1.05,1.68]	1.35*	[1.07,1.71]	1.29*	[1.02,1.64]
Pension	1.13	[0.93,1.38]	1.15	[0.94,1.40]	1.16	[0.95,1.42]	1.12	[0.92,1.36]
Employed	0.7	[0.49,1.02]	0.69*	[0.48,1.00]	0.69*	[0.48,1.00]	0.73	[0.51,1.06]
Unemployed	1	[1.00,1.00]	1	[1.00,1.00]	1	[1.00,1.00]	1	[1.00,1.00]
Homemaker	0.97	[0.72,1.30]	1.04	[0.77,1.39]	1	[0.74,1.34]	1.09	[0.81,1.46]
40-49	1	[1.00,1.00]	1	[1.00,1.00]	1	[1.00,1.00]	1	[1.00,1.00]
50-59	2.31***	[1.47,3.62]	2.39***	[1.52,3.73]	2.34***	[1.49,3.66]	2.28***	[1.46,3.57]
60-69	2.69***	[1.68,4.30]	2.80***	[1.75,4.47]	2.73***	[1.70,4.37]	2.75***	[1.72,4.38]
70-79	3.55***	[2.18,5.78]	3.67***	[2.26,5.96]	3.60***	[2.21,5.87]	3.39***	[2.09,5.52]
80+	6.70***	[4.05,11.07]	6.80***	[4.12,11.22]	7.01***	[4.24,11.58]	5.83***	[3.51,9.67]
HIV Positive	1	[1.00,1.00]	1	[1.00,1.00]	1	[1.00,1.00]	1	[1.00,1.00]
HIV Negative	0.72**	[0.56,0.91]	0.72**	[0.57,0.92]	0.72**	[0.57,0.92]	0.69**	[0.54,0.87]
Missing HIV Data	0.87	[0.52,1.45]	0.81	[0.48,1.35]	0.85	[0.51,1.41]	0.87	[0.52,1.45]
Normal Anemia	1	[1.00,1.00]	1	[1.00,1.00]	1	[1.00,1.00]	1	[1.00,1.00]
Mild Anemia	1.19	[0.95,1.50]	1.17	[0.93,1.47]	1.2	[0.96,1.51]	1.21	[0.97,1.52]
Moderate Anemia	2.02***	[1.58,2.57]	1.96***	[1.54,2.50]	1.99***	[1.56,2.53]	1.93***	[1.52,2.46]
Severe Anemia	3.56***	[2.27,5.58]	3.66***	[2.34,5.75]	3.55***	[2.27,5.57]	3.30***	[2.10,5.17]
Intentional Refusal - Anemia	1.11	[0.47,2.62]	1.24	[0.52,2.93]	1.1	[0.47,2.61]	1.04	[0.44,2.46]
Processing Error - Anemia	1.56*	[1.03,2.38]	1.54*	[1.01,2.34]	1.57*	[1.03,2.39]	1.47	[0.96,2.24]
Hypertensive	1	[1.00,1.00]	1	[1.00,1.00]	1	[1.00,1.00]	1	[1.00,1.00]
Not Hypertensive	0.88	[0.72,1.07]	0.89	[0.73,1.08]	0.88	[0.72,1.08]	0.88	[0.72,1.07]
Intentional Refusal - Hypertension	1.22	[0.64,2.34]	1.28	[0.66,2.45]	1.21	[0.63,2.33]	1.37	[0.71,2.64]
Processing Error - Hypertension	1.65	[0.61,4.48]	1.85	[0.68,5.04]	1.7	[0.62,4.63]	1.83	[0.67,4.97]
Underweight	1.62**	[1.18,2.23]	1.55**	[1.12,2.14]	1.67**	[1.21,2.29]	1.31	[0.93,1.84]

Normal	1	[1.00,1.00]	1	[1.00,1.00]	1	[1.00,1.00]	1	[1.00,1.00]
Overweight	0.87	[0.68,1.11]	0.85	[0.66,1.09]	0.85	[0.66,1.09]	0.89	[0.70,1.14]
Obese	0.79	[0.60,1.04]	0.79	[0.60,1.03]	0.77	[0.59,1.02]	0.82	[0.62,1.07]
Miscellaneous Errors - BMI	3.00***	[2.29,3.94]	2.79***	[2.11,3.69]	2.99***	[2.28,3.93]	2.85**	[1.50,5.42]
No Diabetes/Not Fasting	0.63***	[0.50,0.81]	0.65***	[0.51,0.83]	0.63***	[0.49,0.80]	0.66***	[0.52,0.84]
Diabetic	1	[1.00,1.00]	1	[1.00,1.00]	1	[1.00,1.00]	1	[1.00,1.00]
Not Missing on Diabetes	1.47	[0.72,2.96]	1.51	[0.75,3.05]	1.43	[0.71,2.87]	1.44	[0.71,2.90]
Missing on Diabetes	1	[1.00,1.00]	1	[1.00,1.00]	1	[1.00,1.00]	1	[1.00,1.00]
No Formal Education	1	[1.00,1.00]	1	[1.00,1.00]	1	[1.00,1.00]	1	[1.00,1.00]
Some Primary Education (1-7 years)	1.01	[0.78,1.30]	0.9	[0.73,1.10]	0.87	[0.71,1.07]	0.98	[0.80,1.21]
Some Secondary Education (8-11 years)	1.03	[0.68,1.55]	0.9	[0.63,1.29]	0.85	[0.59,1.23]	1.01	[0.70,1.46]
Secondary Education or more (12+ years)	0.62	[0.34,1.13]	0.57	[0.32,1.01]	0.49*	[0.27,0.88]	0.64	[0.36,1.14]
Born in South Africa	1.2	[0.98,1.47]						
Literacy	0.75*	[0.59,0.96]						
PTSD			0.6	[0.32,1.10]				
Respondent depression			1.07**	[1.02,1.13]				
Father's education					1.05	[0.91,1.22]		
Lowest Wealth					1	[1.00,1.00]		
Low/Mid Wealth					0.93	[0.71,1.21]		
Medium Wealth					1.05	[0.80,1.37]		
Mid/High Wealth					1.01	[0.76,1.34]		
Highest Wealth					1.22	[0.91,1.65]		
Total cognitive score							0.98*	[0.96,1.00]
Missing on cognitive measure							0.52	[0.16,1.68]
Non-Frail							1	[1.00,1.00]
Pre-Frail							1.29*	[1.03,1.61]
Frail							1.98***	[1.33,2.96]
Unable to Score							1.11	[0.59,2.11]
ADL limitation							1.37*	[1.06,1.77]

N for all models is 4907, Exponentiated coefficients; 95% confidence intervals in brackets

* $p<0.05$, ** $p<0.01$, *** $p<0.001$
