# Supplementary material for: Social support receipt as a predictor of mortality: A cohort study in rural South Africa
Source: PLOS Glob Public Health. 2024 Sep 9;4(9):e0003683. doi: 10.1371/journal.pgph.0003683 (PMC11383236; doi:10.1371/journal.pgph.0003683)
Supplement: S4 Table — (PDF) [file pgph.0003683.s004.pdf]

S4 Table: Adjusted Cox Proportional Hazard Models for mortality in HAALSI between waves one and two, by presence of social support domains - (without Health Events).

Support type	Informational		Emotional		Financial		Physical	
A. Main effects only								
Social support	1.09	[0.99,1.18]	1.08	[0.99,1.17]	1.04	[0.95,1.13]	1.06	[0.98,1.16]
B. Sex and support interaction								
Males vs. females	2.14	[1.72,2.66]	2.04	[1.64,2.53]	2.05	[1.65,2.54]	2.03	[1.63,2.51]
Social support in females	1.13	[1.00,1.29]	1.1	[0.97,1.25]	1.1	[0.97,1.25]	1.06	[0.93,1.21]
Social support in males	1.05	[0.94,1.18]	1.06	[0.96,1.18]	1	[0.89,1.12]	1.07	[0.96,1.18]
χ^2 for interaction	0.75		0.17		1.45		0	
p-value	0.39		0.68		0.23		0.99	
C. Age and support interaction								
≥ 60 vs. < 60	6.70	[4.05,11.07]	7.13	[4.33,11.75]	7.01	[4.24,11.58]	7.11	[4.32,11.72]
Social support in those < 60	1.15	[0.97,1.35]	1.1	[0.93,1.30]	0.95	[0.78,1.16]	1.1	[0.92,1.31]
Social support in those ≥ 60	1.06	[0.96,1.18]	1.07	[0.98,1.17]	1.06	[0.97,1.17]	1.06	[0.96,1.16]
χ^2 for interaction	0.58		0.13		1.07		0.14	
p-value	0.45		0.72		0.3		0.7	
