# Supplementary material for: Social support receipt as a predictor of mortality: A cohort study in rural South Africa
Source: PLOS Glob Public Health. 2024 Sep 9;4(9):e0003683. doi: 10.1371/journal.pgph.0003683 (PMC11383236; doi:10.1371/journal.pgph.0003683)
Supplement: S5 Table — (PDF) [file pgph.0003683.s005.pdf]

**S5 Table: Cox Proportional Hazard Models, Full - No Interaction - (without Health Events).**

| | Informational | | Emotional | | Financial | | Physical | |
|---|---|---|---|---|---|---|---|---|
| | Hazard Ratio | Confidence Interval | Hazard Ratio | Confidence Interval | Hazard Ratio | Confidence Interval | Hazard Ratio | Confidence Interval |
| Social support | 1.09 | [0.99,1.18] | 1.08 | [0.99,1.17] | 1.04 | [0.95,1.13] | 1.06 | [0.98,1.16] |
| Sex (Male) | 2.14*** | [1.72,2.66] | 2.04*** | [1.64,2.53] | 2.05*** | [1.65,2.54] | 2.03*** | [1.63,2.51] |
| Never Married | 2.07*** | [1.37,3.13] | 2.14*** | [1.42,3.22] | 2.14*** | [1.42,3.23] | 2.16*** | [1.43,3.25] |
| Married/Partner | 1 | [1.00,1.00] | 1 | [1.00,1.00] | 1 | [1.00,1.00] | 1 | [1.00,1.00] |
| Separated/Deserted/Divorced | 1.46** | [1.10,1.94] | 1.48** | [1.12,1.97] | 1.49** | [1.12,1.98] | 1.49** | [1.12,1.98] |
| Widowed | 1.35* | [1.07,1.70] | 1.35* | [1.07,1.70] | 1.35* | [1.07,1.71] | 1.36* | [1.07,1.72] |
| Pension | 1.13 | [0.93,1.38] | 1.14 | [0.94,1.39] | 1.15 | [0.94,1.41] | 1.14 | [0.94,1.39] |
| Employed | 0.7 | [0.49,1.02] | 0.69* | [0.48,1.00] | 0.69* | [0.47,0.99] | 0.68* | [0.47,0.99] |
| Unemployed | 1 | [1.00,1.00] | 1 | [1.00,1.00] | 1 | [1.00,1.00] | 1 | [1.00,1.00] |
| Homemaker | 0.97 | [0.72,1.30] | 1 | [0.74,1.34] | 1 | [0.74,1.34] | 1.01 | [0.75,1.35] |
| 40-49 | 1 | [1.00,1.00] | 1 | [1.00,1.00] | 1 | [1.00,1.00] | 1 | [1.00,1.00] |
| 50-59 | 2.31*** | [1.47,3.62] | 2.36*** | [1.51,3.70] | 2.33*** | [1.49,3.64] | 2.36*** | [1.51,3.69] |
| 60-69 | 2.68*** | [1.68,4.28] | 2.79*** | [1.75,4.46] | 2.72*** | [1.70,4.35] | 2.80*** | [1.75,4.46] |
| 70-79 | 3.53*** | [2.17,5.74] | 3.70*** | [2.28,6.01] | 3.59*** | [2.20,5.85] | 3.68*** | [2.27,5.99] |
| 80+ | 6.67*** | [4.04,11.01] | 7.12*** | [4.32,11.73] | 7.02*** | [4.25,11.59] | 7.12*** | [4.32,11.73] |
| HIV Positive | 1 | [1.00,1.00] | 1 | [1.00,1.00] | 1 | [1.00,1.00] | 1 | [1.00,1.00] |
| HIV Negative | 0.72** | [0.57,0.91] | 0.73* | [0.58,0.93] | 0.72** | [0.56,0.91] | 0.72** | [0.57,0.92] |
| Missing HIV Data | 0.87 | [0.52,1.45] | 0.85 | [0.51,1.42] | 0.84 | [0.50,1.39] | 0.83 | [0.50,1.39] |
| Normal Anemia | 1 | [1.00,1.00] | 1 | [1.00,1.00] | 1 | [1.00,1.00] | 1 | [1.00,1.00] |
| Mild Anemia | 1.19 | [0.95,1.50] | 1.2 | [0.96,1.50] | 1.2 | [0.96,1.51] | 1.2 | [0.96,1.50] |
| Moderate Anemia | 2.02*** | [1.58,2.57] | 1.98*** | [1.56,2.52] | 1.99*** | [1.56,2.54] | 1.98*** | [1.55,2.52] |
| Severe Anemia | 3.55*** | [2.27,5.57] | 3.56*** | [2.27,5.57] | 3.55*** | [2.27,5.57] | 3.58*** | [2.29,5.60] |
| Intentional Refusal - Anemia | 1.11 | [0.47,2.61] | 1.17 | [0.49,2.77] | 1.13 | [0.48,2.65] | 1.18 | [0.50,2.80] |
| Processing Error - Anemia | 1.55* | [1.02,2.37] | 1.56* | [1.02,2.38] | 1.58* | [1.03,2.40] | 1.57* | [1.03,2.40] |
| Hypertensive | 1 | [1.00,1.00] | 1 | [1.00,1.00] | 1 | [1.00,1.00] | 1 | [1.00,1.00] |
| Not Hypertensive | 0.88 | [0.72,1.07] | 0.88 | [0.72,1.07] | 0.88 | [0.72,1.08] | 0.88 | [0.72,1.08] |
| Intentional Refusal - Hypertension | 1.21 | [0.63,2.32] | 1.24 | [0.65,2.38] | 1.23 | [0.64,2.37] | 1.25 | [0.65,2.40] |
| Processing Error - Hypertension | 1.65 | [0.61,4.49] | 1.69 | [0.62,4.59] | 1.7 | [0.63,4.64] | 1.69 | [0.62,4.58] |
| Underweight | 1.62** | [1.17,2.22] | 1.63** | [1.18,2.24] | 1.66** | [1.21,2.29] | 1.64** | [1.19,2.26] |
| Normal | 1 | [1.00,1.00] | 1 | [1.00,1.00] | 1 | [1.00,1.00] | 1 | [1.00,1.00] |

| | | | | | | | | |
|---|---|---|---|---|---|---|---|---|
| Overweight | 0.87 | [0.68,1.11] | 0.85 | [0.67,1.09] | 0.85 | [0.67,1.09] | 0.86 | [0.67,1.10] |
| Obese | 0.79 | [0.60,1.04] | 0.79 | [0.60,1.03] | 0.77 | [0.59,1.02] | 0.79 | [0.60,1.04] |
| Miscellaneous Errors - BMI | 3.00*** | [2.28,3.93] | 2.99*** | [2.27,3.92] | 3.00*** | [2.29,3.94] | 2.98*** | [2.27,3.91] |
| No Diabetes/Not Fasting | 0.63*** | [0.50,0.81] | 0.63*** | [0.49,0.80] | 0.63*** | [0.49,0.80] | 0.63*** | [0.49,0.80] |
| Diabetic | 1 | [1.00,1.00] | 1 | [1.00,1.00] | 1 | [1.00,1.00] | 1 | [1.00,1.00] |
| Not Missing on Diabetes | 1.45 | [0.72,2.94] | 1.5 | [0.75,3.02] | 1.45 | [0.72,2.92] | 1.5 | [0.74,3.03] |
| Missing on Diabetes | 1 | [1.00,1.00] | 1 | [1.00,1.00] | 1 | [1.00,1.00] | 1 | [1.00,1.00] |
| No Formal Education | 1 | [1.00,1.00] | 1 | [1.00,1.00] | 1 | [1.00,1.00] | 1 | [1.00,1.00] |
| Some Primary Education (1-7 years) | 1.01 | [0.78,1.30] | 0.9 | [0.74,1.11] | 0.87 | [0.71,1.07] | 0.9 | [0.74,1.10] |
| Some Secondary Education (8-11 years) | 1.02 | [0.68,1.53] | 0.9 | [0.63,1.30] | 0.85 | [0.59,1.24] | 0.91 | [0.63,1.30] |
| Secondary Education or more (12+ years) | 0.61 | [0.34,1.12] | 0.55* | [0.31,0.98] | 0.49* | [0.27,0.88] | 0.55* | [0.31,0.97] |
| Born in South Africa | 1.2 | [0.98,1.48] | | | | | | |
| Literacy | 0.75* | [0.58,0.95] | | | | | | |
| Father's education | | | | | 1.06 | [0.91,1.22] | | |
| Lowest Wealth | | | | | 1 | [1.00,1.00] | | |
| Low/Mid Wealth | | | | | 0.93 | [0.71,1.22] | | |
| Medium Wealth | | | | | 1.05 | [0.80,1.37] | | |
| Mid/High Wealth | | | | | 1.01 | [0.76,1.35] | | |
| Highest Wealth | | | | | 1.23 | [0.91,1.65] | | |

N for all models is 4907, Exponentiated coefficients; 95% confidence intervals in brackets

* p<0.05, ** p<0.01, *** p<0.001
