# Supplementary material for: Social support receipt as a predictor of mortality: A cohort study in rural South Africa
Source: PLOS Glob Public Health. 2024 Sep 9;4(9):e0003683. doi: 10.1371/journal.pgph.0003683 (PMC11383236; doi:10.1371/journal.pgph.0003683)
Supplement: S6 Table — (PDF) [file pgph.0003683.s006.pdf]

S6 Table: Cox Proportional Hazard Models, Full - Sex Interaction - (without Health Events).

	Informational		Emotional		Financial		Physical	
	Hazard Ratio	Confidence Interval	Hazard Ratio	Confidence Interval	Hazard Ratio	Confidence Interval	Hazard Ratio	Confidence Interval
Social support x Female	1.13	[1.00,1.29]	1.1	[0.97,1.25]	1.1	[0.97,1.25]	1.06	[0.93,1.21]
Social support x Male	1.05	[0.94,1.18]	1.06	[0.96,1.18]	1	[0.89,1.12]	1.07	[0.96,1.18]
Sex (Male)	2.14***	[1.72,2.66]	2.04***	[1.64,2.53]	2.05***	[1.65,2.54]	2.03***	[1.63,2.51]
Never Married	2.06***	[1.37,3.11]	2.14***	[1.42,3.21]	2.15***	[1.42,3.23]	2.16***	[1.43,3.26]
Married/Partner	1	[1.00,1.00]	1	[1.00,1.00]	1	[1.00,1.00]	1	[1.00,1.00]
Separated/Deserted/Divorced	1.45**	[1.09,1.93]	1.48**	[1.12,1.97]	1.49**	[1.12,1.98]	1.49**	[1.12,1.98]
Widowed	1.34*	[1.06,1.70]	1.35*	[1.07,1.70]	1.34*	[1.06,1.70]	1.36*	[1.07,1.72]
Pension	1.13	[0.93,1.38]	1.14	[0.94,1.39]	1.15	[0.94,1.40]	1.14	[0.94,1.39]
Employed	0.7	[0.48,1.02]	0.69*	[0.47,0.99]	0.69*	[0.47,0.99]	0.68*	[0.47,0.99]
Unemployed	1	[1.00,1.00]	1	[1.00,1.00]	1	[1.00,1.00]	1	[1.00,1.00]
Homemaker	0.97	[0.72,1.30]	1	[0.74,1.33]	1	[0.74,1.34]	1.01	[0.75,1.35]
40-49	1	[1.00,1.00]	1	[1.00,1.00]	1	[1.00,1.00]	1	[1.00,1.00]
50-59	2.31***	[1.48,3.62]	2.36***	[1.51,3.70]	2.32***	[1.48,3.64]	2.36***	[1.51,3.69]
60-69	2.69***	[1.68,4.29]	2.80***	[1.76,4.48]	2.74***	[1.71,4.39]	2.80***	[1.75,4.47]
70-79	3.55***	[2.18,5.78]	3.71***	[2.28,6.03]	3.62***	[2.22,5.90]	3.68***	[2.26,5.99]
80+	6.70***	[4.06,11.08]	7.15***	[4.34,11.78]	7.05***	[4.27,11.65]	7.12***	[4.32,11.73]
HIV Positive	1	[1.00,1.00]	1	[1.00,1.00]	1	[1.00,1.00]	1	[1.00,1.00]
HIV Negative	0.72**	[0.57,0.91]	0.73*	[0.58,0.93]	0.72**	[0.56,0.91]	0.72**	[0.57,0.92]
Missing HIV Data	0.87	[0.53,1.45]	0.85	[0.51,1.42]	0.84	[0.50,1.39]	0.83	[0.50,1.39]
Normal Anemia	1	[1.00,1.00]	1	[1.00,1.00]	1	[1.00,1.00]	1	[1.00,1.00]
Mild Anemia	1.19	[0.95,1.50]	1.2	[0.95,1.50]	1.2	[0.96,1.50]	1.2	[0.96,1.50]
Moderate Anemia	2.01***	[1.58,2.56]	1.98***	[1.55,2.52]	1.98***	[1.56,2.53]	1.98***	[1.55,2.52]
Severe Anemia	3.59***	[2.29,5.63]	3.58***	[2.28,5.60]	3.62***	[2.30,5.68]	3.58***	[2.28,5.61]
Intentional Refusal - Anemia	1.1	[0.46,2.59]	1.17	[0.49,2.76]	1.11	[0.47,2.64]	1.18	[0.50,2.80]
Processing Error - Anemia	1.56*	[1.02,2.37]	1.56*	[1.03,2.38]	1.58*	[1.04,2.41]	1.57*	[1.03,2.40]
Hypertensive	1	[1.00,1.00]	1	[1.00,1.00]	1	[1.00,1.00]	1	[1.00,1.00]
Not Hypertensive	0.88	[0.72,1.07]	0.88	[0.72,1.07]	0.88	[0.72,1.07]	0.88	[0.72,1.08]
Intentional Refusal - Hypertension	1.2	[0.63,2.30]	1.24	[0.65,2.38]	1.24	[0.64,2.39]	1.25	[0.65,2.40]
Processing Error - Hypertension	1.63	[0.60,4.44]	1.68	[0.62,4.57]	1.7	[0.62,4.62]	1.69	[0.62,4.58]
Underweight	1.62**	[1.18,2.24]	1.63**	[1.19,2.25]	1.67**	[1.21,2.30]	1.64**	[1.19,2.26]

Normal	1	[1.00,1.00]	1	[1.00,1.00]	1	[1.00,1.00]	1	[1.00,1.00]
Overweight	0.87	[0.68,1.11]	0.86	[0.67,1.09]	0.86	[0.67,1.10]	0.86	[0.67,1.10]
Obese	0.79	[0.60,1.04]	0.79	[0.60,1.03]	0.77	[0.59,1.02]	0.79	[0.60,1.04]
Miscellaneous Errors - BMI	3.02***	[2.30,3.96]	2.99***	[2.28,3.92]	3.01***	[2.29,3.95]	2.98***	[2.27,3.91]
No Diabetes/Not Fasting	0.63***	[0.50,0.81]	0.63***	[0.49,0.80]	0.63***	[0.50,0.81]	0.63***	[0.49,0.80]
Diabetic	1	[1.00,1.00]	1	[1.00,1.00]	1	[1.00,1.00]	1	[1.00,1.00]
Not Missing on Diabetes	1.45	[0.71,2.94]	1.5	[0.74,3.02]	1.45	[0.72,2.92]	1.5	[0.74,3.03]
Missing on Diabetes	1	[1.00,1.00]	1	[1.00,1.00]	1	[1.00,1.00]	1	[1.00,1.00]
No Formal Education	1	[1.00,1.00]	1	[1.00,1.00]	1	[1.00,1.00]	1	[1.00,1.00]
Some Primary Education (1-7 years)	1.01	[0.78,1.30]	0.9	[0.74,1.11]	0.87	[0.71,1.07]	0.9	[0.74,1.10]
Some Secondary Education (8-11 years)	1.02	[0.68,1.54]	0.91	[0.63,1.30]	0.85	[0.59,1.24]	0.91	[0.63,1.30]
Secondary Education or more (12+ years)	0.62	[0.34,1.12]	0.55*	[0.31,0.98]	0.49*	[0.27,0.88]	0.55*	[0.31,0.97]
Born in South Africa	1.21	[0.98,1.48]						
Literacy	0.75*	[0.59,0.96]						
Father's education					1.06	[0.91,1.22]		
Lowest Wealth					1	[1.00,1.00]		
Low/Mid Wealth					0.94	[0.72,1.23]		
Medium Wealth					1.05	[0.80,1.38]		
Mid/High Wealth					1.02	[0.77,1.36]		
Highest Wealth					1.23	[0.91,1.66]		

N for all models is 4907, Exponentiated coefficients; 95% confidence intervals in brackets

* p<0.05, ** p<0.01, *** p<0.001
