# Supplementary material for: Social support receipt as a predictor of mortality: A cohort study in rural South Africa
Source: PLOS Glob Public Health. 2024 Sep 9;4(9):e0003683. doi: 10.1371/journal.pgph.0003683 (PMC11383236; doi:10.1371/journal.pgph.0003683)
Supplement: S7 Table — (PDF) [file pgph.0003683.s007.pdf]

**S7 Table: Cox Proportional Hazard Models, Full - Age Interaction - (without Health Events).**

| | Informational | | Emotional | | Financial | | Physical | |
|---|---|---|---|---|---|---|---|---|
| | Hazard Ratio | Confidence Interval | Hazard Ratio | Confidence Interval | Hazard Ratio | Confidence Interval | Hazard Ratio | Confidence Interval |
| Social support x Under 60 | 1.15 | [0.97,1.35] | 1.1 | [0.93,1.30] | 0.95 | [0.78,1.16] | 1.1 | [0.92,1.31] |
| Social support x Older/Equal to 60 | 1.06 | [0.96,1.18] | 1.07 | [0.98,1.17] | 1.06 | [0.97,1.17] | 1.06 | [0.96,1.16] |
| Sex (Male) | 2.14*** | [1.72,2.66] | 2.04*** | [1.64,2.53] | 2.04*** | [1.65,2.54] | 2.03*** | [1.63,2.52] |
| Never Married | 2.09*** | [1.38,3.15] | 2.14*** | [1.42,3.22] | 2.14*** | [1.42,3.22] | 2.17*** | [1.44,3.27] |
| Married/Partner | 1 | [1.00,1.00] | 1 | [1.00,1.00] | 1 | [1.00,1.00] | 1 | [1.00,1.00] |
| Separated/Deserted/Divorced | 1.46** | [1.10,1.94] | 1.48** | [1.12,1.97] | 1.49** | [1.12,1.98] | 1.49** | [1.12,1.98] |
| Widowed | 1.34* | [1.06,1.70] | 1.35* | [1.07,1.70] | 1.35* | [1.07,1.71] | 1.36* | [1.07,1.72] |
| Pension | 1.13 | [0.93,1.38] | 1.14 | [0.94,1.39] | 1.16 | [0.95,1.42] | 1.14 | [0.93,1.39] |
| Employed | 0.7 | [0.49,1.02] | 0.69* | [0.47,1.00] | 0.69* | [0.48,1.00] | 0.68* | [0.47,0.99] |
| Unemployed | 1 | [1.00,1.00] | 1 | [1.00,1.00] | 1 | [1.00,1.00] | 1 | [1.00,1.00] |
| Homemaker | 0.97 | [0.72,1.30] | 1 | [0.74,1.34] | 1 | [0.74,1.34] | 1.01 | [0.75,1.36] |
| 40-49 | 1 | [1.00,1.00] | 1 | [1.00,1.00] | 1 | [1.00,1.00] | 1 | [1.00,1.00] |
| 50-59 | 2.31*** | [1.47,3.62] | 2.36*** | [1.51,3.69] | 2.34*** | [1.49,3.66] | 2.35*** | [1.50,3.68] |
| 60-69 | 2.69*** | [1.68,4.30] | 2.80*** | [1.75,4.47] | 2.73*** | [1.70,4.37] | 2.80*** | [1.75,4.46] |
| 70-79 | 3.55*** | [2.18,5.78] | 3.70*** | [2.28,6.02] | 3.60*** | [2.21,5.87] | 3.68*** | [2.27,5.99] |
| 80+ | 6.70*** | [4.05,11.07] | 7.13*** | [4.33,11.75] | 7.01*** | [4.24,11.58] | 7.11*** | [4.32,11.72] |
| HIV Positive | 1 | [1.00,1.00] | 1 | [1.00,1.00] | 1 | [1.00,1.00] | 1 | [1.00,1.00] |
| HIV Negative | 0.72** | [0.56,0.91] | 0.73* | [0.57,0.93] | 0.72** | [0.57,0.92] | 0.72** | [0.57,0.92] |
| Missing HIV Data | 0.87 | [0.52,1.45] | 0.85 | [0.51,1.42] | 0.85 | [0.51,1.41] | 0.83 | [0.50,1.39] |
| Normal Anemia | 1 | [1.00,1.00] | 1 | [1.00,1.00] | 1 | [1.00,1.00] | 1 | [1.00,1.00] |
| Mild Anemia | 1.19 | [0.95,1.50] | 1.2 | [0.95,1.50] | 1.2 | [0.96,1.51] | 1.2 | [0.96,1.50] |
| Moderate Anemia | 2.02*** | [1.58,2.57] | 1.98*** | [1.56,2.52] | 1.99*** | [1.56,2.53] | 1.98*** | [1.56,2.52] |
| Severe Anemia | 3.56*** | [2.27,5.58] | 3.57*** | [2.28,5.59] | 3.55*** | [2.27,5.57] | 3.58*** | [2.29,5.61] |
| Intentional Refusal - Anemia | 1.11 | [0.47,2.62] | 1.17 | [0.50,2.77] | 1.1 | [0.47,2.61] | 1.18 | [0.50,2.80] |
| Processing Error - Anemia | 1.56* | [1.03,2.38] | 1.56* | [1.03,2.38] | 1.57* | [1.03,2.39] | 1.58* | [1.04,2.40] |
| Hypertensive | 1 | [1.00,1.00] | 1 | [1.00,1.00] | 1 | [1.00,1.00] | 1 | [1.00,1.00] |
| Not Hypertensive | 0.88 | [0.72,1.07] | 0.88 | [0.72,1.07] | 0.88 | [0.72,1.08] | 0.88 | [0.72,1.07] |
| Intentional Refusal - Hypertension | 1.22 | [0.64,2.34] | 1.25 | [0.65,2.39] | 1.21 | [0.63,2.33] | 1.25 | [0.65,2.41] |
| Processing Error - Hypertension | 1.65 | [0.61,4.48] | 1.69 | [0.62,4.58] | 1.7 | [0.62,4.63] | 1.69 | [0.62,4.58] |
| Underweight | 1.62** | [1.18,2.23] | 1.63** | [1.18,2.25] | 1.67** | [1.21,2.29] | 1.64** | [1.19,2.26] |
| Normal | 1 | [1.00,1.00] | 1 | [1.00,1.00] | 1 | [1.00,1.00] | 1 | [1.00,1.00] |

| | | | | | | | | |
|---|---|---|---|---|---|---|---|---|
| Overweight | 0.87 | [0.68,1.11] | 0.86 | [0.67,1.09] | 0.85 | [0.66,1.09] | 0.86 | [0.67,1.10] |
| Obese | 0.79 | [0.60,1.04] | 0.79 | [0.60,1.03] | 0.77 | [0.59,1.02] | 0.79 | [0.60,1.04] |
| Miscellaneous Errors - BMI | 3.00*** | [2.29,3.94] | 2.99*** | [2.28,3.92] | 2.99*** | [2.28,3.93] | 2.99*** | [2.28,3.92] |
| No Diabetes/Not Fasting | 0.63*** | [0.50,0.81] | 0.63*** | [0.49,0.80] | 0.63*** | [0.49,0.80] | 0.63*** | [0.49,0.80] |
| Diabetic | 1 | [1.00,1.00] | 1 | [1.00,1.00] | 1 | [1.00,1.00] | 1 | [1.00,1.00] |
| Not Missing on Diabetes | 1.47 | [0.72,2.96] | 1.51 | [0.75,3.03] | 1.43 | [0.71,2.87] | 1.51 | [0.75,3.05] |
| Missing on Diabetes | 1 | [1.00,1.00] | 1 | [1.00,1.00] | 1 | [1.00,1.00] | 1 | [1.00,1.00] |
| No Formal Education | 1 | [1.00,1.00] | 1 | [1.00,1.00] | 1 | [1.00,1.00] | 1 | [1.00,1.00] |
| Some Primary Education (1-7 years) | 1.01 | [0.78,1.30] | 0.9 | [0.74,1.11] | 0.87 | [0.71,1.07] | 0.9 | [0.74,1.10] |
| Some Secondary Education (8-11 years) | 1.03 | [0.68,1.55] | 0.91 | [0.63,1.30] | 0.85 | [0.59,1.23] | 0.91 | [0.63,1.31] |
| Secondary Education or more (12+ years) | 0.62 | [0.34,1.13] | 0.55* | [0.31,0.98] | 0.49* | [0.27,0.88] | 0.55* | [0.31,0.97] |
| Born in South Africa | 1.2 | [0.98,1.47] | | | | | | |
| Literacy | 0.75* | [0.59,0.96] | | | | | | |
| Father's education | | | | | 1.05 | [0.91,1.22] | | |
| Lowest Wealth | | | | | 1 | [1.00,1.00] | | |
| Low/Mid Wealth | | | | | 0.93 | [0.71,1.21] | | |
| Medium Wealth | | | | | 1.05 | [0.80,1.37] | | |
| Mid/High Wealth | | | | | 1.01 | [0.76,1.34] | | |
| Highest Wealth | | | | | 1.22 | [0.91,1.65] | | |

N for all models is 4907, Exponentiated coefficients; 95% confidence intervals in brackets

* p<0.05, ** p<0.01, *** p<0.001
