# Supplementary material for: Social support receipt as a predictor of mortality: A cohort study in rural South Africa
Source: PLOS Glob Public Health. 2024 Sep 9;4(9):e0003683. doi: 10.1371/journal.pgph.0003683 (PMC11383236; doi:10.1371/journal.pgph.0003683)
Supplement: S8 Table — (PDF) [file pgph.0003683.s008.pdf]

S8 Table: Adjusted Accelerated Failure Time Hazard Models for mortality in HAALSI between waves one and two, by presence of social support domains

**Support type**	**Informational**		**Emotional**		**Financial**		**Physical**	
A. Main effects only								
Social support	1.09	[0.99,1.18]	1.08	[1.00,1.17]	1.04	[0.96,1.13]	1.07	[0.98,1.16]
B. Sex and support interaction								
Males vs. females	2.12	[1.71,2.64]	2.04	[1.64,2.52]	2.03	[1.64,2.52]	2.05	[1.64,2.54]
Social support in females	1.13	[0.99,1.29]	1.1	[0.97,1.25]	1.1	[0.97,1.24]	1.06	[0.93,1.21]
Social support in males	1.05	[0.94,1.18]	1.07	[0.96,1.19]	1	[0.89,1.12]	1.08	[0.97,1.20]
χ^2 for interaction	0.7		0.11		1.36		0.04	
p-value	0.4		0.74		0.24		0.84	
C. Age and support interaction								
≥ 60 vs. < 60	6.61	[4.00,10.93]	6.72	[4.07,11.08]	6.91	[4.18,11.43]	5.75	[3.47,9.54]
Social support in those < 60	1.15	[0.98,1.35]	1.11	[0.93,1.31]	0.95	[0.78,1.16]	1.11	[0.93,1.32]
Social support in those ≥ 60	1.06	[0.96,1.18]	1.08	[0.98,1.18]	1.06	[0.97,1.17]	1.06	[0.96,1.16]
χ^2 for interaction	0.61		0.09		1.04		0.2	
p-value	0.43		0.77		0.31		0.66	
