# Supplementary material for: Social support receipt as a predictor of mortality: A cohort study in rural South Africa
Source: PLOS Glob Public Health. 2024 Sep 9;4(9):e0003683. doi: 10.1371/journal.pgph.0003683 (PMC11383236; doi:10.1371/journal.pgph.0003683)
Supplement: S9 Table — (PDF) [file pgph.0003683.s009.pdf]

S9 Table: Accelerated Failure Time Hazard Models, No Interaction

	Informational		Emotional		Financial		Physical	
	Hazard Ratio	Confidence Interval	Hazard Ratio	Confidence Interval	Hazard Ratio	Confidence Interval	Hazard Ratio	Confidence Interval
Social support	1.09	[0.99,1.18]	1.08	[1.00,1.17]	1.04	[0.96,1.13]	1.04	[0.96,1.13]
Sex (Male)	2.12***	[1.71,2.64]	2.03***	[1.64,2.52]	2.03***	[1.64,2.53]	2.03***	[1.64,2.53]
Never Married	2.06***	[1.36,3.10]	2.11***	[1.40,3.18]	2.13***	[1.41,3.21]	2.13***	[1.41,3.21]
Married/Partner	1	[1.00,1.00]	1	[1.00,1.00]	1	[1.00,1.00]	1	[1.00,1.00]
Separated/Deserted/Divorced	1.46**	[1.10,1.94]	1.45*	[1.09,1.92]	1.49**	[1.12,1.98]	1.49**	[1.12,1.98]
Widowed	1.35*	[1.07,1.70]	1.33*	[1.05,1.68]	1.35*	[1.07,1.71]	1.35*	[1.07,1.71]
Pension	1.13	[0.93,1.38]	1.15	[0.94,1.40]	1.15	[0.94,1.41]	1.15	[0.94,1.41]
Employed	0.7	[0.49,1.02]	0.69	[0.48,1.00]	0.69*	[0.48,1.00]	0.69*	[0.48,1.00]
Unemployed	1	[1.00,1.00]	1	[1.00,1.00]	1	[1.00,1.00]	1	[1.00,1.00]
Homemaker	0.97	[0.72,1.30]	1.03	[0.77,1.39]	1	[0.74,1.34]	1	[0.74,1.34]
40-49	1	[1.00,1.00]	1	[1.00,1.00]	1	[1.00,1.00]	1	[1.00,1.00]
50-59	2.30***	[1.47,3.60]	2.37***	[1.52,3.72]	2.32***	[1.48,3.63]	2.32***	[1.48,3.63]
60-69	2.67***	[1.67,4.27]	2.79***	[1.75,4.45]	2.72***	[1.70,4.35]	2.72***	[1.70,4.35]
70-79	3.51***	[2.16,5.71]	3.64***	[2.24,5.91]	3.57***	[2.19,5.82]	3.57***	[2.19,5.82]
80+	6.58***	[3.99,10.87]	6.70***	[4.07,11.05]	6.92***	[4.19,11.44]	6.92***	[4.19,11.44]
HIV Positive	1	[1.00,1.00]	1	[1.00,1.00]	1	[1.00,1.00]	1	[1.00,1.00]
HIV Negative	0.72**	[0.57,0.92]	0.73**	[0.57,0.92]	0.72**	[0.57,0.92]	0.72**	[0.57,0.92]
Missing HIV Data	0.88	[0.53,1.47]	0.82	[0.49,1.37]	0.85	[0.51,1.41]	0.85	[0.51,1.41]
Normal Anemia	1	[1.00,1.00]	1	[1.00,1.00]	1	[1.00,1.00]	1	[1.00,1.00]
Mild Anemia	1.19	[0.95,1.50]	1.17	[0.93,1.47]	1.2	[0.96,1.51]	1.2	[0.96,1.51]
Moderate Anemia	2.00***	[1.57,2.55]	1.95***	[1.53,2.48]	1.98***	[1.55,2.52]	1.98***	[1.55,2.52]
Severe Anemia	3.46***	[2.21,5.42]	3.54***	[2.26,5.55]	3.46***	[2.21,5.42]	3.46***	[2.21,5.42]
Intentional Refusal - Anemia	1.08	[0.46,2.56]	1.2	[0.51,2.87]	1.1	[0.47,2.59]	1.1	[0.47,2.59]
Processing Error - Anemia	1.55*	[1.02,2.36]	1.54*	[1.01,2.34]	1.57*	[1.03,2.40]	1.57*	[1.03,2.40]
Hypertensive	1	[1.00,1.00]	1	[1.00,1.00]	1	[1.00,1.00]	1	[1.00,1.00]
Not Hypertensive	0.88	[0.72,1.07]	0.89	[0.73,1.08]	0.88	[0.72,1.08]	0.88	[0.72,1.08]
Intentional Refusal - Hypertension	1.23	[0.64,2.34]	1.28	[0.67,2.46]	1.24	[0.65,2.39]	1.24	[0.65,2.39]
Processing Error - Hypertension	1.63	[0.60,4.42]	1.82	[0.67,4.95]	1.67	[0.61,4.56]	1.67	[0.61,4.56]
Underweight	1.62**	[1.17,2.22]	1.55**	[1.12,2.14]	1.66**	[1.21,2.29]	1.66**	[1.21,2.29]
Normal	1	[1.00,1.00]	1	[1.00,1.00]	1	[1.00,1.00]	1	[1.00,1.00]

Overweight	0.87	[0.68,1.11]	0.85	[0.66,1.09]	0.85	[0.67,1.09]	0.85	[0.67,1.09]
Obese	0.79	[0.60,1.04]	0.79	[0.60,1.03]	0.77	[0.59,1.02]	0.77	[0.59,1.02]
Miscellaneous Errors - BMI	2.95***	[2.25,3.87]	2.75***	[2.08,3.62]	2.96***	[2.25,3.88]	2.96***	[2.25,3.88]
No Diabetes/Not Fasting	0.63***	[0.50,0.81]	0.65***	[0.51,0.84]	0.63***	[0.49,0.81]	0.63***	[0.49,0.81]
Diabetic	1	[1.00,1.00]	1	[1.00,1.00]	1	[1.00,1.00]	1	[1.00,1.00]
Not Missing on Diabetes	1.44	[0.71,2.91]	1.49	[0.74,3.01]	1.44	[0.71,2.89]	1.44	[0.71,2.89]
Missing on Diabetes	1	[1.00,1.00]	1	[1.00,1.00]	1	[1.00,1.00]	1	[1.00,1.00]
No Formal Education	1	[1.00,1.00]	1	[1.00,1.00]	1	[1.00,1.00]	1	[1.00,1.00]
Some Primary Education (1-7 years)	1.01	[0.78,1.30]	0.9	[0.74,1.10]	0.88	[0.71,1.08]	0.88	[0.71,1.08]
Some Secondary Education (8-11 years)	1.01	[0.67,1.53]	0.9	[0.63,1.29]	0.85	[0.59,1.23]	0.85	[0.59,1.23]
Secondary Education or more (12+ years)	0.62	[0.34,1.13]	0.57	[0.32,1.02]	0.49*	[0.27,0.89]	0.49*	[0.27,0.89]
Born in South Africa	1.2	[0.98,1.48]						
Literacy	0.75*	[0.59,0.96]						
Emotional support								
PTSD			0.6	[0.33,1.11]				
Respondent depression			1.07**	[1.02,1.13]				
Father's education					1.06	[0.91,1.22]		
Lowest Wealth					1	[1.00,1.00]		
Low/Mid Wealth					0.93	[0.72,1.22]		
Medium Wealth					1.04	[0.80,1.37]		
Mid/High Wealth					1.01	[0.76,1.34]		
Highest Wealth					1.22	[0.91,1.65]		
Total cognitive score							0.98	[0.96,1.00]
Missing on cognitive measure							0.52	[0.16,1.68]
Non-Frail							1	[1.00,1.00]
Pre-Frail							1.28*	[1.02,1.61]
Frail							1.98***	[1.32,2.95]
Unable to Score							1.11	[0.58,2.10]
ADL limitation							1.37*	[1.06,1.76]

N for all models is 4907, Exponentiated coefficients; 95% confidence intervals in brackets

* $p<0.05$, ** $p<0.01$, *** $p<0.001$
