# Supplementary material for: Social support receipt as a predictor of mortality: A cohort study in rural South Africa
Source: PLOS Glob Public Health. 2024 Sep 9;4(9):e0003683. doi: 10.1371/journal.pgph.0003683 (PMC11383236; doi:10.1371/journal.pgph.0003683)
Supplement: S10 Table — (PDF) [file pgph.0003683.s010.pdf]

S10 Table: Accelerated Failure Time Hazard Models, Sex Interaction

	Informational		Emotional		Financial		Physical	
	Hazard Ratio	Confidence Interval	Hazard Ratio	Confidence Interval	Hazard Ratio	Confidence Interval	Hazard Ratio	Confidence Interval
Social support x Female	1.13	[0.99,1.29]	1.1	[0.97,1.25]	1.1	[0.97,1.24]	1.06	[0.93,1.21]
Social support x Male	1.05	[0.94,1.18]	1.07	[0.96,1.19]	1	[0.89,1.12]	1.08	[0.97,1.20]
Sex (Male)	2.12***	[1.71,2.64]	2.04***	[1.64,2.52]	2.03***	[1.64,2.52]	2.05***	[1.64,2.54]
Never Married	2.04***	[1.36,3.08]	2.11***	[1.40,3.17]	2.13***	[1.41,3.21]	2.02***	[1.34,3.06]
Married/Partner	1	[1.00,1.00]	1	[1.00,1.00]	1	[1.00,1.00]	1	[1.00,1.00]
Separated/Deserted/Divorced	1.45**	[1.09,1.93]	1.44*	[1.09,1.92]	1.49**	[1.12,1.98]	1.46**	[1.10,1.94]
Widowed	1.34*	[1.06,1.70]	1.33*	[1.05,1.68]	1.34*	[1.06,1.70]	1.30*	[1.02,1.64]
Pension	1.13	[0.93,1.38]	1.15	[0.94,1.40]	1.15	[0.94,1.40]	1.12	[0.92,1.36]
Employed	0.7	[0.49,1.02]	0.69	[0.48,1.00]	0.69*	[0.47,0.99]	0.73	[0.51,1.07]
Unemployed	1	[1.00,1.00]	1	[1.00,1.00]	1	[1.00,1.00]	1	[1.00,1.00]
Homemaker	0.97	[0.72,1.30]	1.03	[0.77,1.39]	1	[0.74,1.34]	1.08	[0.80,1.45]
40-49	1	[1.00,1.00]	1	[1.00,1.00]	1	[1.00,1.00]	1	[1.00,1.00]
50-59	2.30***	[1.47,3.60]	2.38***	[1.52,3.72]	2.32***	[1.48,3.63]	2.28***	[1.46,3.57]
60-69	2.68***	[1.68,4.28]	2.79***	[1.75,4.46]	2.74***	[1.71,4.38]	2.74***	[1.72,4.37]
70-79	3.53***	[2.17,5.74]	3.65***	[2.25,5.92]	3.60***	[2.21,5.87]	3.37***	[2.07,5.48]
80+	6.62***	[4.01,10.93]	6.72***	[4.08,11.09]	6.95***	[4.21,11.49]	5.75***	[3.47,9.54]
HIV Positive	1	[1.00,1.00]	1	[1.00,1.00]	1	[1.00,1.00]	1	[1.00,1.00]
HIV Negative	0.72**	[0.57,0.92]	0.73**	[0.57,0.92]	0.72**	[0.57,0.92]	0.69**	[0.54,0.88]
Missing HIV Data	0.89	[0.53,1.48]	0.82	[0.49,1.37]	0.85	[0.51,1.42]	0.88	[0.53,1.47]
Normal Anemia	1	[1.00,1.00]	1	[1.00,1.00]	1	[1.00,1.00]	1	[1.00,1.00]
Mild Anemia	1.19	[0.95,1.50]	1.17	[0.93,1.47]	1.2	[0.96,1.50]	1.21	[0.97,1.52]
Moderate Anemia	2.00***	[1.57,2.54]	1.94***	[1.53,2.47]	1.97***	[1.54,2.51]	1.92***	[1.50,2.44]
Severe Anemia	3.49***	[2.23,5.47]	3.55***	[2.26,5.57]	3.52***	[2.24,5.52]	3.20***	[2.04,5.03]
Intentional Refusal - Anemia	1.07	[0.45,2.54]	1.2	[0.51,2.87]	1.09	[0.46,2.58]	1.02	[0.43,2.42]
Processing Error - Anemia	1.56*	[1.02,2.37]	1.54*	[1.01,2.34]	1.58*	[1.04,2.40]	1.46	[0.96,2.24]
Hypertensive	1	[1.00,1.00]	1	[1.00,1.00]	1	[1.00,1.00]	1	[1.00,1.00]
Not Hypertensive	0.88	[0.72,1.07]	0.89	[0.73,1.08]	0.88	[0.72,1.07]	0.88	[0.72,1.07]
Intentional Refusal - Hypertension	1.22	[0.64,2.33]	1.28	[0.67,2.46]	1.25	[0.65,2.41]	1.38	[0.72,2.66]
Processing Error - Hypertension	1.61	[0.59,4.38]	1.82	[0.67,4.94]	1.67	[0.61,4.55]	1.78	[0.66,4.85]
Underweight	1.62**	[1.18,2.24]	1.55**	[1.12,2.14]	1.67**	[1.21,2.30]	1.31	[0.93,1.84]

Normal	1	[1.00,1.00]	1	[1.00,1.00]	1	[1.00,1.00]	1	[1.00,1.00]
Overweight	0.87	[0.68,1.11]	0.85	[0.66,1.09]	0.86	[0.67,1.10]	0.89	[0.70,1.14]
Obese	0.79	[0.60,1.04]	0.79	[0.60,1.03]	0.77	[0.59,1.02]	0.82	[0.62,1.07]
Miscellaneous Errors - BMI	2.96***	[2.26,3.89]	2.75***	[2.08,3.63]	2.96***	[2.25,3.89]	2.81**	[1.48,5.35]
No Diabetes/Not Fasting	0.63***	[0.50,0.81]	0.65***	[0.51,0.84]	0.63***	[0.50,0.81]	0.66***	[0.52,0.85]
Diabetic	1	[1.00,1.00]	1	[1.00,1.00]	1	[1.00,1.00]	1	[1.00,1.00]
Not Missing on Diabetes	1.43	[0.70,2.91]	1.48	[0.73,3.01]	1.43	[0.71,2.89]	1.41	[0.70,2.85]
Missing on Diabetes	1	[1.00,1.00]	1	[1.00,1.00]	1	[1.00,1.00]	1	[1.00,1.00]
No Formal Education	1	[1.00,1.00]	1	[1.00,1.00]	1	[1.00,1.00]	1	[1.00,1.00]
Some Primary Education (1-7 years)	1.01	[0.78,1.30]	0.9	[0.74,1.10]	0.88	[0.71,1.08]	0.99	[0.80,1.22]
Some Secondary Education (8-11 years)	1.02	[0.68,1.53]	0.9	[0.63,1.29]	0.85	[0.59,1.24]	1	[0.69,1.45]
Secondary Education or more (12+ years)	0.62	[0.34,1.13]	0.58	[0.33,1.02]	0.49*	[0.27,0.89]	0.64	[0.36,1.15]
Born in South Africa	1.21	[0.98,1.48]						
Literacy	0.75*	[0.59,0.96]						
PTSD			0.6	[0.33,1.11]				
Respondent depression			1.07**	[1.02,1.13]				
Father's education					1.06	[0.91,1.22]		
Lowest Wealth					1	[1.00,1.00]		
Low/Mid Wealth					0.94	[0.72,1.23]		
Medium Wealth					1.05	[0.80,1.37]		
Mid/High Wealth					1.02	[0.77,1.35]		
Highest Wealth					1.23	[0.91,1.66]		
Total cognitive score							0.98*	[0.96,1.00]
Missing on cognitive measure							0.52	[0.16,1.68]
Non-Frail							1	[1.00,1.00]
Pre-Frail							1.28*	[1.02,1.61]
Frail							1.98***	[1.33,2.96]
Unable to Score							1.11	[0.58,2.10]
ADL limitation							1.36*	[1.06,1.76]

N for all models is 4907, Exponentiated coefficients; 95% confidence intervals in brackets

* p<0.05, ** p<0.01, *** p<0.001
