# Supplementary material for: Social support receipt as a predictor of mortality: A cohort study in rural South Africa
Source: PLOS Glob Public Health. 2024 Sep 9;4(9):e0003683. doi: 10.1371/journal.pgph.0003683 (PMC11383236; doi:10.1371/journal.pgph.0003683)
Supplement: S12 Table — (PDF) [file pgph.0003683.s012.pdf]

S12 Table: Adjusted Cox Proportional Hazard Models for mortality in HAALSI between waves one and two, by presence of social support domains - (Dichotomous Support).

Support type	Informational		Emotional		Financial		Physical	
A. Main effects only								
Social support	1.31	[0.93,1.85]	1.2	[0.87,1.64]	0.86	[0.69,1.08]	1.27	[0.93,1.74]
B. Sex and support interaction								
Males vs. females	2.50	[1.28,4.87]	2.16	[1.19,3.92]	2.67	[1.81,3.94]	2.00	[1.11,3.61]
Social support in females	1.43	[0.85,2.40]	1.23	[0.79,1.93]	1.06	[0.76,1.48]	1.25	[0.82,1.90]
Social support in males	1.21	[0.76,1.92]	1.16	[0.75,1.79]	0.74	[0.55,0.98]	1.30	[0.82,2.05]
χ^2 for interaction	0.22		0.04		2.81		0.02	
p-value	0.64		0.85		0.09		0.89	
C. Age and support interaction								
≥ 60 vs. < 60	14.01	[4.91,39.98]	14.71	[5.51,39.28]	5.50	[2.96,10.20]	6.30	[2.77,14.32]
Social support in those < 60	2.48	[1.00,6.14]	2.30	[1.00,5.27]	0.66	[0.43,1.00]	1.34	[0.71,2.52]
Social support in those ≥ 60	1.13	[0.78,1.64]	1.03	[0.73,1.45]	0.94	[0.73,1.21]	1.25	[0.88,1.78]
χ^2 for interaction	2.53		3.14		2.15		0.04	
p-value	0.11		0.08		0.14		0.85	
