# Supplementary material for: Social support receipt as a predictor of mortality: A cohort study in rural South Africa
Source: PLOS Glob Public Health. 2024 Sep 9;4(9):e0003683. doi: 10.1371/journal.pgph.0003683 (PMC11383236; doi:10.1371/journal.pgph.0003683)
Supplement: S13 Table — (PDF) [file pgph.0003683.s013.pdf]

S13 Table: Cox Proportional Hazard Models, Full - No Interaction - (Dichotomous Support).

	Informational		Emotional		Financial		Physical	
	Hazard Ratio	Confidence Interval	Hazard Ratio	Confidence Interval	Hazard Ratio	Confidence Interval	Hazard Ratio	Confidence Interval
> one month of Social Support	1.31	[0.93,1.85]	1.2	[0.87,1.64]	0.86	[0.69,1.08]	1.27	[0.93,1.74]
Sex (Male)	2.15***	[1.73,2.68]	2.05***	[1.65,2.54]	2.03***	[1.63,2.52]	2.08***	[1.67,2.58]
Never Married	2.09***	[1.39,3.16]	2.13***	[1.42,3.22]	2.09***	[1.38,3.15]	2.07***	[1.37,3.13]
Married/Partner	1	[1.00,1.00]	1	[1.00,1.00]	1	[1.00,1.00]	1	[1.00,1.00]
Separated/Deserted/Divorced	1.44*	[1.09,1.91]	1.42*	[1.07,1.88]	1.44*	[1.08,1.91]	1.45**	[1.09,1.93]
Widowed	1.35*	[1.06,1.70]	1.32*	[1.05,1.67]	1.29*	[1.01,1.63]	1.30*	[1.03,1.65]
Pension	1.12	[0.92,1.37]	1.14	[0.93,1.39]	1.13	[0.92,1.37]	1.11	[0.91,1.35]
Employed	0.7	[0.48,1.01]	0.69*	[0.48,1.00]	0.68*	[0.47,0.99]	0.73	[0.50,1.06]
Unemployed	1	[1.00,1.00]	1	[1.00,1.00]	1	[1.00,1.00]	1	[1.00,1.00]
Homemaker	1	[0.75,1.34]	1.08	[0.80,1.46]	0.96	[0.71,1.29]	1.09	[0.81,1.47]
40-49	1	[1.00,1.00]	1	[1.00,1.00]	1	[1.00,1.00]	1	[1.00,1.00]
50-59	2.30***	[1.47,3.60]	2.39***	[1.53,3.75]	2.34***	[1.50,3.67]	2.30***	[1.47,3.60]
60-69	2.70***	[1.69,4.32]	2.85***	[1.78,4.54]	2.76***	[1.73,4.42]	2.79***	[1.75,4.45]
70-79	3.56***	[2.19,5.80]	3.73***	[2.30,6.07]	3.65***	[2.24,5.95]	3.44***	[2.12,5.60]
80+	6.71***	[4.06,11.08]	6.94***	[4.21,11.44]	7.25***	[4.39,11.95]	5.92***	[3.57,9.81]
HIV Positive	1	[1.00,1.00]	1	[1.00,1.00]	1	[1.00,1.00]	1	[1.00,1.00]
HIV Negative	0.71**	[0.56,0.90]	0.71**	[0.56,0.90]	0.72**	[0.56,0.91]	0.69**	[0.54,0.87]
Missing HIV Data	0.86	[0.51,1.42]	0.81	[0.49,1.35]	0.85	[0.51,1.41]	0.88	[0.53,1.46]
Normal Anemia	1	[1.00,1.00]	1	[1.00,1.00]	1	[1.00,1.00]	1	[1.00,1.00]
Mild Anemia	1.2	[0.96,1.51]	1.17	[0.93,1.47]	1.19	[0.95,1.50]	1.22	[0.97,1.53]
Moderate Anemia	2.02***	[1.59,2.57]	1.96***	[1.54,2.49]	1.99***	[1.56,2.53]	1.93***	[1.51,2.45]
Severe Anemia	3.48***	[2.22,5.45]	3.60***	[2.30,5.65]	3.48***	[2.22,5.46]	3.26***	[2.08,5.12]
Intentional Refusal - Anemia	1.06	[0.45,2.49]	1.16	[0.49,2.74]	1.08	[0.46,2.56]	1	[0.42,2.35]
Processing Error - Anemia	1.55*	[1.02,2.35]	1.54*	[1.01,2.34]	1.58*	[1.04,2.40]	1.46	[0.95,2.23]
Hypertensive	1	[1.00,1.00]	1	[1.00,1.00]	1	[1.00,1.00]	1	[1.00,1.00]
Not Hypertensive	0.88	[0.72,1.08]	0.9	[0.73,1.09]	0.88	[0.72,1.08]	0.88	[0.72,1.08]
Intentional Refusal - Hypertension	1.21	[0.63,2.31]	1.28	[0.66,2.45]	1.22	[0.64,2.36]	1.37	[0.71,2.64]
Processing Error - Hypertension	1.57	[0.58,4.28]	1.78	[0.65,4.84]	1.75	[0.64,4.76]	1.74	[0.64,4.74]
Underweight	1.63**	[1.19,2.25]	1.57**	[1.14,2.17]	1.69**	[1.22,2.32]	1.32	[0.94,1.85]
Normal	1	[1.00,1.00]	1	[1.00,1.00]	1	[1.00,1.00]	1	[1.00,1.00]

Overweight	0.88	[0.69,1.12]	0.85	[0.67,1.09]	0.85	[0.66,1.09]	0.9	[0.70,1.15]
Obese	0.8	[0.61,1.05]	0.79	[0.60,1.04]	0.77	[0.59,1.01]	0.82	[0.62,1.07]
Miscellaneous Errors - BMI	3.04***	[2.32,3.99]	2.82***	[2.14,3.73]	3.00***	[2.29,3.95]	2.90**	[1.53,5.53]
No Diabetes/Not Fasting	0.63***	[0.49,0.81]	0.65***	[0.51,0.84]	0.63***	[0.49,0.81]	0.65***	[0.51,0.84]
Diabetic	1	[1.00,1.00]	1	[1.00,1.00]	1	[1.00,1.00]	1	[1.00,1.00]
Not Missing on Diabetes	1.38	[0.68,2.79]	1.45	[0.72,2.93]	1.45	[0.72,2.94]	1.38	[0.68,2.77]
Missing on Diabetes	1	[1.00,1.00]	1	[1.00,1.00]	1	[1.00,1.00]	1	[1.00,1.00]
No Formal Education	1	[1.00,1.00]	1	[1.00,1.00]	1	[1.00,1.00]	1	[1.00,1.00]
Some Primary Education (1-7 years)	1.03	[0.80,1.33]	0.9	[0.74,1.10]	0.87	[0.71,1.07]	0.99	[0.80,1.22]
Some Secondary Education (8-11 years)	1.03	[0.69,1.55]	0.91	[0.63,1.31]	0.86	[0.59,1.25]	1.01	[0.70,1.47]
Secondary Education or more (12+ years)	0.62	[0.34,1.13]	0.57	[0.32,1.01]	0.49*	[0.27,0.88]	0.63	[0.35,1.13]
Born in South Africa	1.19	[0.97,1.46]						
Literacy	0.74*	[0.58,0.95]						
PTSD			0.6	[0.33,1.11]				
Respondent depression			1.07**	[1.02,1.13]				
Father's education					1.04	[0.90,1.21]		
Lowest Wealth					1	[1.00,1.00]		
Low/Mid Wealth					0.94	[0.72,1.22]		
Medium Wealth					1.06	[0.81,1.39]		
Mid/High Wealth					1.02	[0.77,1.35]		
Highest Wealth					1.24	[0.92,1.68]		
Total cognitive score							0.98*	[0.96,1.00]
Missing on cognitive measure							0.52	[0.16,1.69]
Non-Frail							1	[1.00,1.00]
Pre-Frail							1.27*	[1.02,1.60]
Frail							1.96***	[1.31,2.93]
Unable to Score							1.09	[0.57,2.06]
ADL limitation							1.39*	[1.08,1.79]

N for all models is 4907, Exponentiated coefficients; 95% confidence intervals in brackets
* $p<0.05$, ** $p<0.01$, *** $p<0.001$
