# Supplementary material for: Social support receipt as a predictor of mortality: A cohort study in rural South Africa
Source: PLOS Glob Public Health. 2024 Sep 9;4(9):e0003683. doi: 10.1371/journal.pgph.0003683 (PMC11383236; doi:10.1371/journal.pgph.0003683)
Supplement: S14 Table — (PDF) [file pgph.0003683.s014.pdf]

**S14 Table: Cox Proportional Hazard Models, Full - Sex Interaction - (Dichotomous Support).**

| | Informational | | Emotional | | Financial | | Physical | |
|---|---|---|---|---|---|---|---|---|
| | Hazard Ratio | Confidence Interval | Hazard Ratio | Confidence Interval | Hazard Ratio | Confidence Interval | Hazard Ratio | Confidence Interval |
| Sex (Male) | 2.50** | [1.28,4.87] | 2.16* | [1.19,3.92] | 2.67*** | [1.81,3.94] | 2.00* | [1.11,3.61] |
| > one month of Social Support x Female | 1.43 | [0.85,2.40] | 1.23 | [0.79,1.93] | 1.06 | [0.76,1.48] | 1.25 | [0.82,1.90] |
| > one month of Social Support x Male | 1.21 | [0.76,1.92] | 1.16 | [0.75,1.79] | 0.74* | [0.55,0.98] | 1.3 | [0.82,2.05] |
| Never Married | 2.07*** | [1.37,3.14] | 2.13*** | [1.41,3.21] | 2.05*** | [1.36,3.10] | 2.08*** | [1.37,3.15] |
| Married/Partner | 1 | [1.00,1.00] | 1 | [1.00,1.00] | 1 | [1.00,1.00] | 1 | [1.00,1.00] |
| Separated/Deserted/Divorced | 1.44* | [1.08,1.90] | 1.42* | [1.07,1.88] | 1.42* | [1.07,1.89] | 1.46** | [1.09,1.94] |
| Widowed | 1.34* | [1.06,1.70] | 1.32* | [1.04,1.67] | 1.27 | [1.00,1.60] | 1.30* | [1.03,1.65] |
| Pension | 1.12 | [0.92,1.37] | 1.14 | [0.93,1.39] | 1.13 | [0.92,1.37] | 1.11 | [0.91,1.35] |
| Employed | 0.7 | [0.48,1.01] | 0.69* | [0.48,1.00] | 0.68* | [0.47,0.99] | 0.73 | [0.50,1.06] |
| Unemployed | 1 | [1.00,1.00] | 1 | [1.00,1.00] | 1 | [1.00,1.00] | 1 | [1.00,1.00] |
| Homemaker | 1 | [0.75,1.35] | 1.08 | [0.80,1.46] | 0.95 | [0.71,1.28] | 1.09 | [0.81,1.47] |
| 40-49 | 1 | [1.00,1.00] | 1 | [1.00,1.00] | 1 | [1.00,1.00] | 1 | [1.00,1.00] |
| 50-59 | 2.30*** | [1.47,3.61] | 2.39*** | [1.53,3.74] | 2.35*** | [1.50,3.67] | 2.30*** | [1.47,3.60] |
| 60-69 | 2.71*** | [1.70,4.33] | 2.85*** | [1.78,4.54] | 2.78*** | [1.74,4.44] | 2.79*** | [1.75,4.45] |
| 70-79 | 3.57*** | [2.19,5.82] | 3.73*** | [2.30,6.07] | 3.68*** | [2.26,5.99] | 3.44*** | [2.12,5.60] |
| 80+ | 6.73*** | [4.07,11.12] | 6.95*** | [4.21,11.46] | 7.27*** | [4.41,12.00] | 5.92*** | [3.57,9.81] |
| HIV Positive | 1 | [1.00,1.00] | 1 | [1.00,1.00] | 1 | [1.00,1.00] | 1 | [1.00,1.00] |
| HIV Negative | 0.71** | [0.56,0.90] | 0.71** | [0.56,0.90] | 0.72** | [0.57,0.91] | 0.69** | [0.54,0.87] |
| Missing HIV Data | 0.86 | [0.52,1.43] | 0.81 | [0.49,1.36] | 0.86 | [0.52,1.44] | 0.87 | [0.53,1.46] |
| Normal Anemia | 1 | [1.00,1.00] | 1 | [1.00,1.00] | 1 | [1.00,1.00] | 1 | [1.00,1.00] |
| Mild Anemia | 1.2 | [0.96,1.50] | 1.17 | [0.93,1.47] | 1.19 | [0.95,1.49] | 1.22 | [0.97,1.53] |
| Moderate Anemia | 2.02*** | [1.59,2.57] | 1.96*** | [1.54,2.49] | 1.99*** | [1.56,2.53] | 1.93*** | [1.51,2.45] |
| Severe Anemia | 3.47*** | [2.21,5.43] | 3.59*** | [2.29,5.64] | 3.45*** | [2.20,5.42] | 3.26*** | [2.08,5.13] |
| Intentional Refusal - Anemia | 1.06 | [0.45,2.50] | 1.15 | [0.49,2.74] | 1.05 | [0.44,2.50] | 1 | [0.42,2.35] |
| Processing Error - Anemia | 1.55* | [1.02,2.36] | 1.54* | [1.01,2.34] | 1.58* | [1.04,2.40] | 1.46 | [0.95,2.23] |
| Hypertensive | 1 | [1.00,1.00] | 1 | [1.00,1.00] | 1 | [1.00,1.00] | 1 | [1.00,1.00] |
| Not Hypertensive | 0.88 | [0.72,1.07] | 0.9 | [0.73,1.09] | 0.88 | [0.72,1.08] | 0.88 | [0.72,1.08] |
| Intentional Refusal - Hypertension | 1.21 | [0.63,2.32] | 1.27 | [0.66,2.45] | 1.23 | [0.64,2.37] | 1.37 | [0.71,2.64] |
| Processing Error - Hypertension | 1.58 | [0.58,4.31] | 1.78 | [0.66,4.85] | 1.8 | [0.66,4.90] | 1.74 | [0.64,4.73] |
| Underweight | 1.64** | [1.19,2.25] | 1.57** | [1.14,2.17] | 1.69** | [1.22,2.32] | 1.32 | [0.94,1.85] |

| | | | | | | | | |
|---|---|---|---|---|---|---|---|---|
| Normal | 1 | [1.00,1.00] | 1 | [1.00,1.00] | 1 | [1.00,1.00] | 1 | [1.00,1.00] |
| Overweight | 0.88 | [0.69,1.12] | 0.85 | [0.67,1.09] | 0.85 | [0.66,1.09] | 0.9 | [0.70,1.15] |
| Obese | 0.8 | [0.61,1.05] | 0.79 | [0.60,1.04] | 0.77 | [0.58,1.01] | 0.82 | [0.62,1.07] |
| Miscellaneous Errors - BMI | 3.04*** | [2.32,4.00] | 2.83*** | [2.14,3.73] | 2.98*** | [2.27,3.92] | 2.91** | [1.53,5.53] |
| No Diabetes/Not Fasting | 0.63*** | [0.50,0.81] | 0.65*** | [0.51,0.84] | 0.64*** | [0.50,0.81] | 0.65*** | [0.51,0.84] |
| Diabetic | 1 | [1.00,1.00] | 1 | [1.00,1.00] | 1 | [1.00,1.00] | 1 | [1.00,1.00] |
| Not Missing on Diabetes | 1.39 | [0.68,2.81] | 1.45 | [0.72,2.92] | 1.43 | [0.71,2.90] | 1.38 | [0.68,2.76] |
| Missing on Diabetes | 1 | [1.00,1.00] | 1 | [1.00,1.00] | 1 | [1.00,1.00] | 1 | [1.00,1.00] |
| No Formal Education | 1 | [1.00,1.00] | 1 | [1.00,1.00] | 1 | [1.00,1.00] | 1 | [1.00,1.00] |
| Some Primary Education (1-7 years) | 1.03 | [0.80,1.32] | 0.9 | [0.74,1.10] | 0.86 | [0.70,1.06] | 0.99 | [0.80,1.22] |
| Some Secondary Education (8-11 years) | 1.03 | [0.69,1.55] | 0.91 | [0.63,1.31] | 0.86 | [0.59,1.25] | 1.01 | [0.70,1.47] |
| Secondary Education or more (12+ years) | 0.62 | [0.34,1.13] | 0.57 | [0.32,1.01] | 0.49* | [0.27,0.89] | 0.63 | [0.35,1.13] |
| Born in South Africa | 1.19 | [0.97,1.46] | | | | | | |
| Literacy | 0.74* | [0.58,0.95] | | | | | | |
| PTSD | | | 0.6 | [0.33,1.11] | | | | |
| Respondent depression | | | 1.07** | [1.02,1.13] | | | | |
| Father's education | | | | | 1.04 | [0.90,1.21] | | |
| Lowest Wealth | | | | | 1 | [1.00,1.00] | | |
| Low/Mid Wealth | | | | | 0.94 | [0.72,1.22] | | |
| Medium Wealth | | | | | 1.06 | [0.81,1.38] | | |
| Mid/High Wealth | | | | | 1.02 | [0.77,1.35] | | |
| Highest Wealth | | | | | 1.24 | [0.92,1.67] | | |
| Total cognitive score | | | | | | | 0.98* | [0.96,1.00] |
| Missing on cognitive measure | | | | | | | 0.52 | [0.16,1.69] |
| Non-Frail | | | | | | | 1 | [1.00,1.00] |
| Pre-Frail | | | | | | | 1.27* | [1.02,1.60] |
| Frail | | | | | | | 1.96*** | [1.31,2.93] |
| Unable to Score | | | | | | | 1.09 | [0.57,2.06] |
| ADL limitation | | | | | | | 1.39* | [1.08,1.79] |

N for all models is 4907, Exponentiated coefficients; 95% confidence intervals in brackets
* p<0.05, ** p<0.01, *** p<0.001
