# Supplementary material for: Social support receipt as a predictor of mortality: A cohort study in rural South Africa
Source: PLOS Glob Public Health. 2024 Sep 9;4(9):e0003683. doi: 10.1371/journal.pgph.0003683 (PMC11383236; doi:10.1371/journal.pgph.0003683)
Supplement: S15 Table — (PDF) [file pgph.0003683.s015.pdf]

S15 Table: Cox Proportional Hazard Models, Full - Age Interaction - (Dichotomous Support)

	Informational		Emotional		Financial		Physical	
	Hazard Ratio	Confidence Interval	Hazard Ratio	Confidence Interval	Hazard Ratio	Confidence Interval	Hazard Ratio	Confidence Interval
> one month of Social Support x < 60	2.48*	[1.00,6.14]	2.30*	[1.00,5.27]	0.66*	[0.43,1.00]	1.34	[0.71,2.52]
> one month of Social Support x ≥ 60	1.13	[0.78,1.64]	1.03	[0.73,1.45]	0.94	[0.73,1.21]	1.25	[0.88,1.78]
Sex (Male)	2.15***	[1.73,2.68]	2.07***	[1.67,2.57]	2.02***	[1.62,2.50]	2.08***	[1.67,2.58]
Never Married	2.15***	[1.43,3.24]	2.18***	[1.45,3.28]	2.07***	[1.37,3.12]	2.07***	[1.37,3.14]
Married/Partner	1	[1.00,1.00]	1	[1.00,1.00]	1	[1.00,1.00]	1	[1.00,1.00]
Separated/Deserted/Divorced	1.45*	[1.09,1.92]	1.43*	[1.08,1.89]	1.42*	[1.06,1.89]	1.45**	[1.09,1.93]
Widowed	1.34*	[1.06,1.70]	1.32*	[1.05,1.67]	1.28*	[1.01,1.63]	1.30*	[1.03,1.65]
Pension	1.13	[0.93,1.37]	1.15	[0.94,1.40]	1.13	[0.93,1.38]	1.11	[0.91,1.35]
Employed	0.69	[0.48,1.00]	0.68*	[0.47,0.98]	0.69*	[0.47,0.99]	0.73	[0.50,1.06]
Unemployed	1	[1.00,1.00]	1	[1.00,1.00]	1	[1.00,1.00]	1	[1.00,1.00]
Homemaker	1	[0.75,1.34]	1.07	[0.79,1.45]	0.96	[0.71,1.29]	1.09	[0.81,1.47]
40-49	1	[1.00,1.00]	1	[1.00,1.00]	1	[1.00,1.00]	1	[1.00,1.00]
50-59	2.27***	[1.45,3.56]	2.40***	[1.54,3.76]	2.37***	[1.51,3.71]	2.30***	[1.47,3.59]
60-69	5.63**	[2.01,15.79]	6.00***	[2.29,15.71]	2.11*	[1.18,3.79]	2.97**	[1.34,6.57]
70-79	7.43***	[2.62,21.05]	7.88***	[2.98,20.83]	2.79***	[1.53,5.09]	3.67**	[1.63,8.24]
80+	14.01***	[4.91,39.98]	14.71***	[5.51,39.28]	5.50***	[2.96,10.20]	6.30***	[2.77,14.32]
HIV Positive	1	[1.00,1.00]	1	[1.00,1.00]	1	[1.00,1.00]	1	[1.00,1.00]
HIV Negative	0.71**	[0.56,0.90]	0.71**	[0.56,0.90]	0.72**	[0.57,0.91]	0.69**	[0.54,0.87]
Missing HIV Data	0.85	[0.51,1.42]	0.8	[0.48,1.34]	0.85	[0.51,1.41]	0.87	[0.53,1.46]
Normal Anemia	1	[1.00,1.00]	1	[1.00,1.00]	1	[1.00,1.00]	1	[1.00,1.00]
Mild Anemia	1.2	[0.95,1.50]	1.17	[0.93,1.46]	1.2	[0.95,1.50]	1.22	[0.97,1.53]
Moderate Anemia	2.02***	[1.59,2.57]	1.97***	[1.55,2.50]	1.99***	[1.56,2.53]	1.93***	[1.51,2.45]
Severe Anemia	3.51***	[2.24,5.49]	3.65***	[2.33,5.73]	3.48***	[2.22,5.47]	3.27***	[2.08,5.13]
Intentional Refusal - Anemia	1.08	[0.46,2.56]	1.17	[0.49,2.77]	1.07	[0.45,2.53]	1	[0.42,2.35]
Processing Error - Anemia	1.54*	[1.01,2.35]	1.53*	[1.01,2.33]	1.58*	[1.04,2.40]	1.46	[0.95,2.23]
Hypertensive	1	[1.00,1.00]	1	[1.00,1.00]	1	[1.00,1.00]	1	[1.00,1.00]
Not Hypertensive	0.88	[0.72,1.07]	0.89	[0.73,1.09]	0.88	[0.72,1.08]	0.88	[0.72,1.08]
Intentional Refusal - Hypertension	1.22	[0.64,2.34]	1.32	[0.69,2.53]	1.18	[0.61,2.29]	1.37	[0.71,2.65]
Processing Error - Hypertension	1.57	[0.58,4.27]	1.78	[0.65,4.83]	1.73	[0.63,4.72]	1.74	[0.64,4.75]
Underweight	1.63**	[1.19,2.25]	1.58**	[1.14,2.17]	1.70**	[1.23,2.34]	1.32	[0.94,1.85]

Normal	1	[1.00,1.00]	1	[1.00,1.00]	1	[1.00,1.00]	1	[1.00,1.00]
Overweight	0.88	[0.69,1.13]	0.85	[0.67,1.09]	0.85	[0.66,1.09]	0.9	[0.70,1.15]
Obese	0.8	[0.61,1.04]	0.79	[0.60,1.03]	0.77	[0.59,1.02]	0.82	[0.62,1.07]
Miscellaneous Errors - BMI	3.01***	[2.29,3.96]	2.80***	[2.12,3.70]	3.02***	[2.30,3.97]	2.90**	[1.52,5.52]
No Diabetes/Not Fasting	0.63***	[0.49,0.80]	0.65***	[0.51,0.83]	0.63***	[0.50,0.81]	0.66***	[0.51,0.84]
Diabetic	1	[1.00,1.00]	1	[1.00,1.00]	1	[1.00,1.00]	1	[1.00,1.00]
Not Missing on Diabetes	1.4	[0.69,2.83]	1.46	[0.72,2.95]	1.42	[0.70,2.86]	1.38	[0.68,2.77]
Missing on Diabetes	1	[1.00,1.00]	1	[1.00,1.00]	1	[1.00,1.00]	1	[1.00,1.00]
No Formal Education	1	[1.00,1.00]	1	[1.00,1.00]	1	[1.00,1.00]	1	[1.00,1.00]
Some Primary Education (1-7 years)	1.04	[0.80,1.34]	0.91	[0.74,1.11]	0.87	[0.71,1.07]	0.99	[0.80,1.22]
Some Secondary Education (8-11 years)	1.04	[0.69,1.56]	0.92	[0.64,1.31]	0.86	[0.59,1.25]	1.01	[0.70,1.47]
Secondary Education or more (12+ years)	0.62	[0.34,1.13]						
Born in South Africa	1.19	[0.97,1.46]						
Literacy	0.74*	[0.58,0.94]						
PTSD			0.6	[0.33,1.11]				
Respondent depression			1.07**	[1.02,1.13]				
Father's education					1.04	[0.90,1.21]		
Lowest Wealth					1	[1.00,1.00]		
Low/Mid Wealth					0.94	[0.72,1.23]		
Medium Wealth					1.05	[0.81,1.38]		
Mid/High Wealth					1.02	[0.77,1.35]		
Highest Wealth					1.24	[0.92,1.67]		
Total cognitive score							0.98*	[0.96,1.00]
Missing on cognitive measure							0.52	[0.16,1.69]
Non-Frail							1	[1.00,1.00]
Pre-Frail							1.27*	[1.02,1.60]
Frail							1.96***	[1.31,2.93]
Unable to Score							1.09	[0.57,2.07]
ADL limitation							1.39*	[1.08,1.79]

N for all models is 4907, Exponentiated coefficients; 95% confidence intervals in brackets

* p<0.05, ** p<0.01, *** p<0.001
