# Supplementary figures and images for: Social support receipt as a predictor of mortality: A cohort study in rural South Africa
Source: PLOS Glob Public Health. 2024 Sep 9;4(9):e0003683. doi: 10.1371/journal.pgph.0003683 (PMC11383236; doi:10.1371/journal.pgph.0003683)

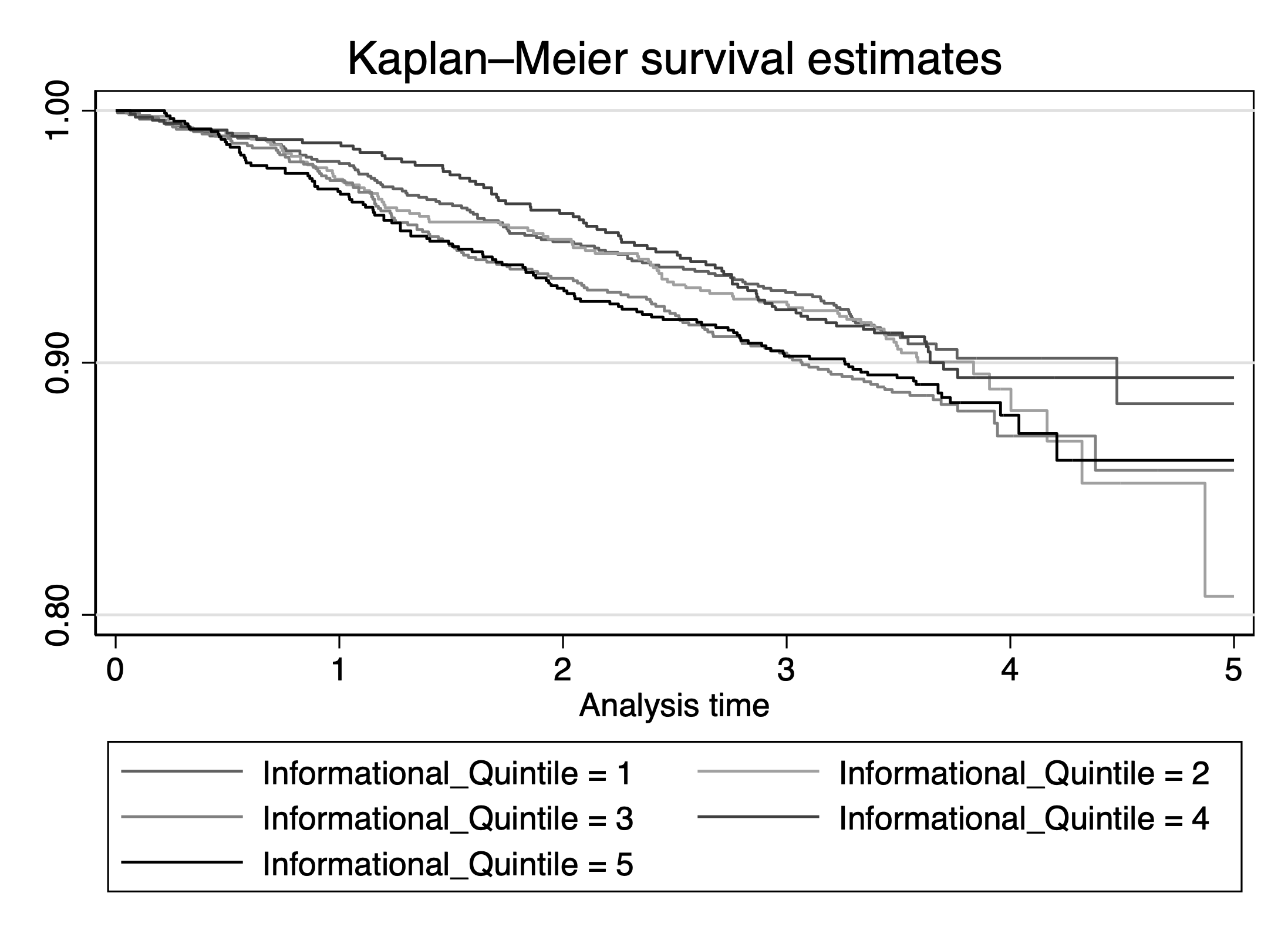

Supplement: S1 Fig — (TIF) [file pgph.0003683.s016.tif]

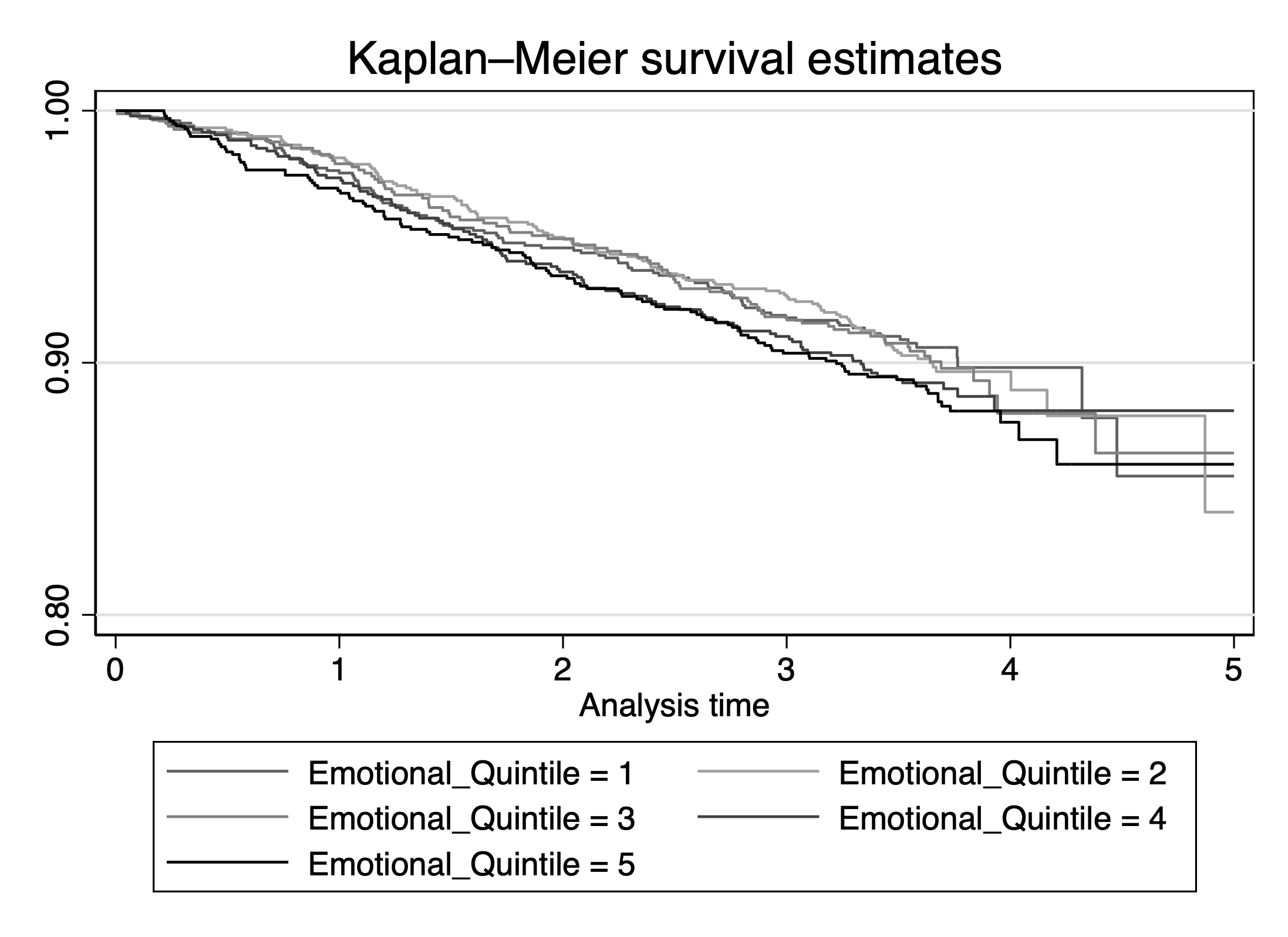

Supplement: S2 Fig — (TIF) [file pgph.0003683.s017.tif]

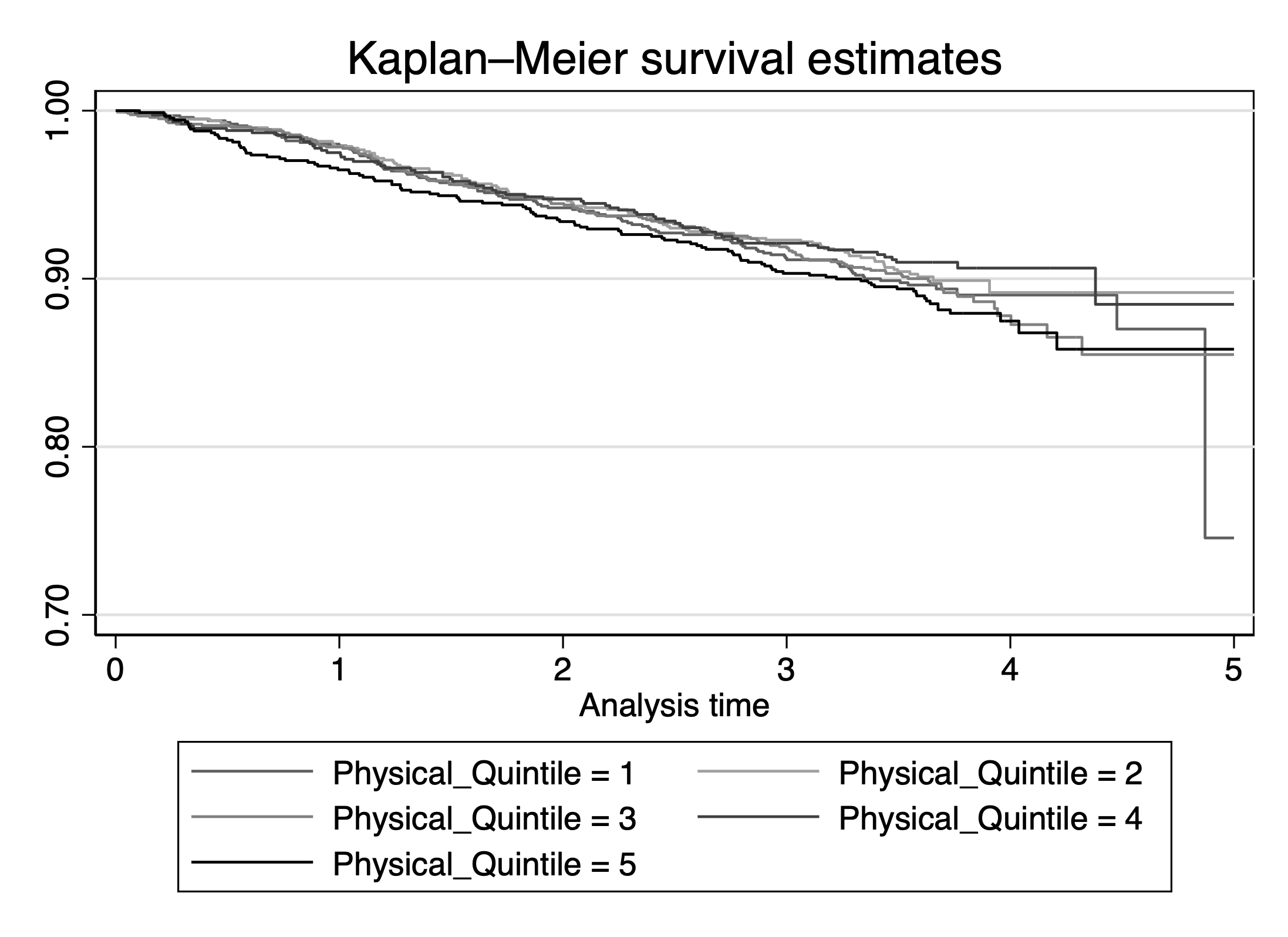

Supplement: S3 Fig — (TIF) [file pgph.0003683.s018.tif]

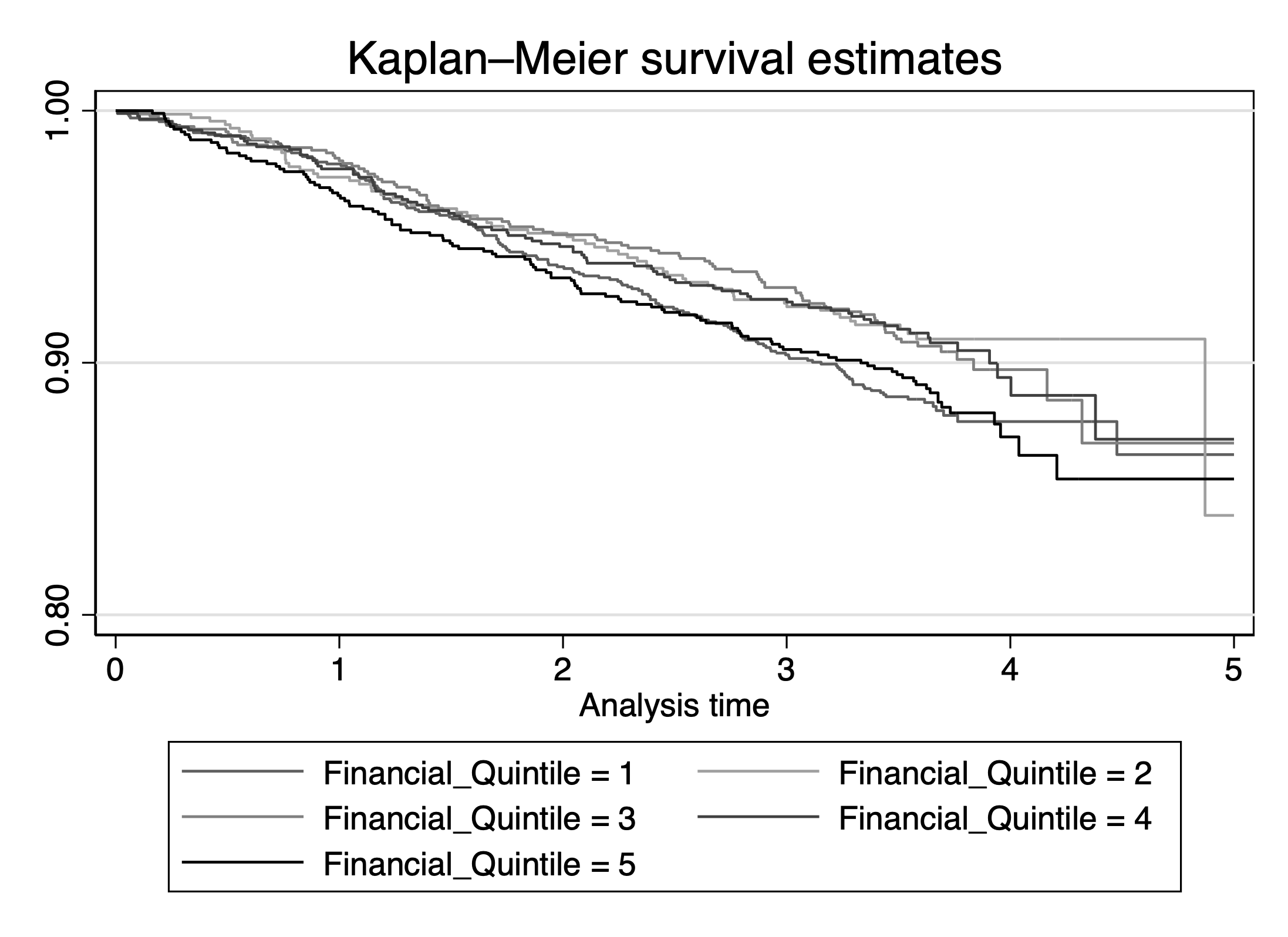

Supplement: S4 Fig — (TIF) [file pgph.0003683.s019.tif]

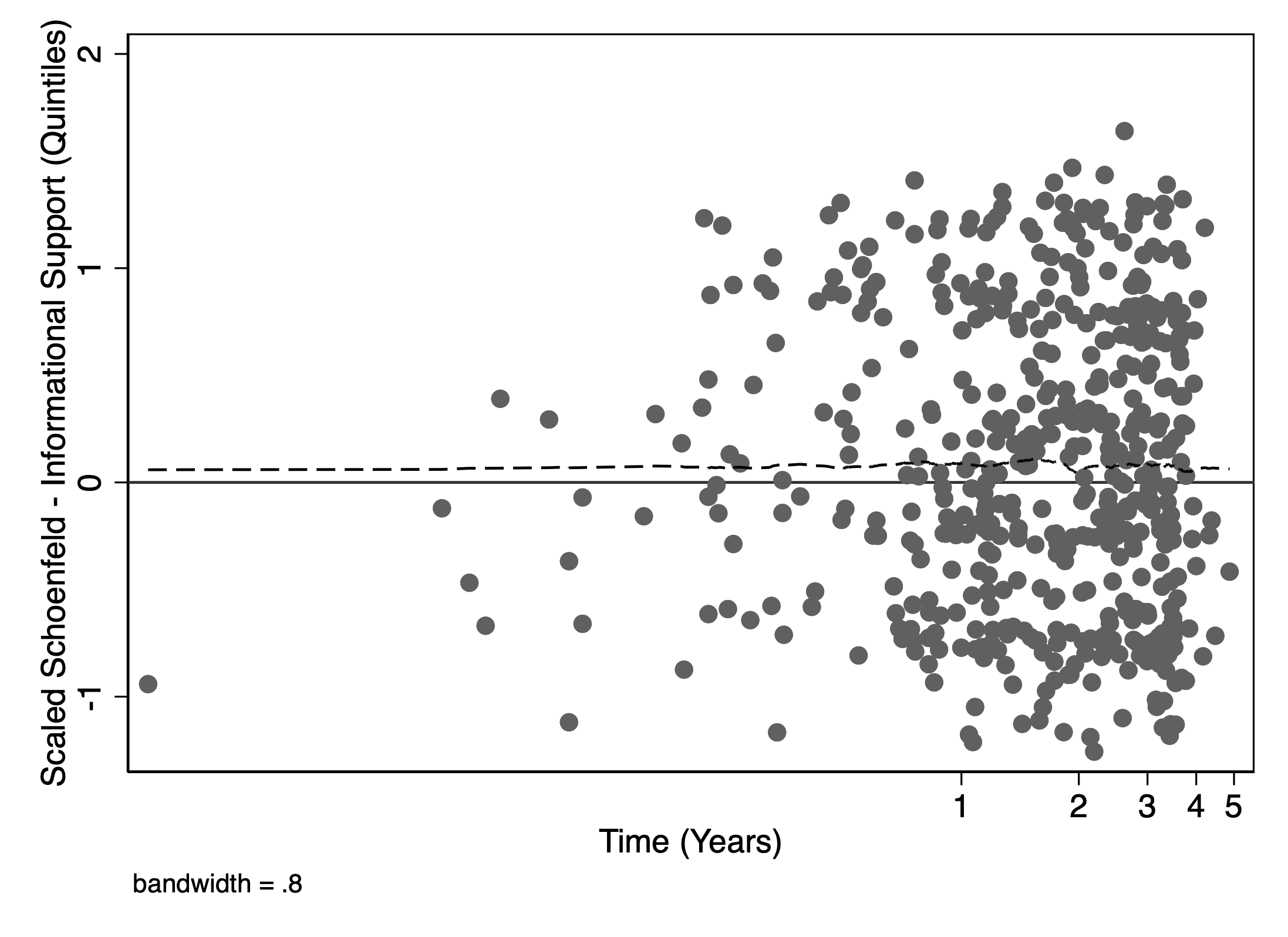

Supplement: S5 Fig — (TIF) [file pgph.0003683.s020.tif]

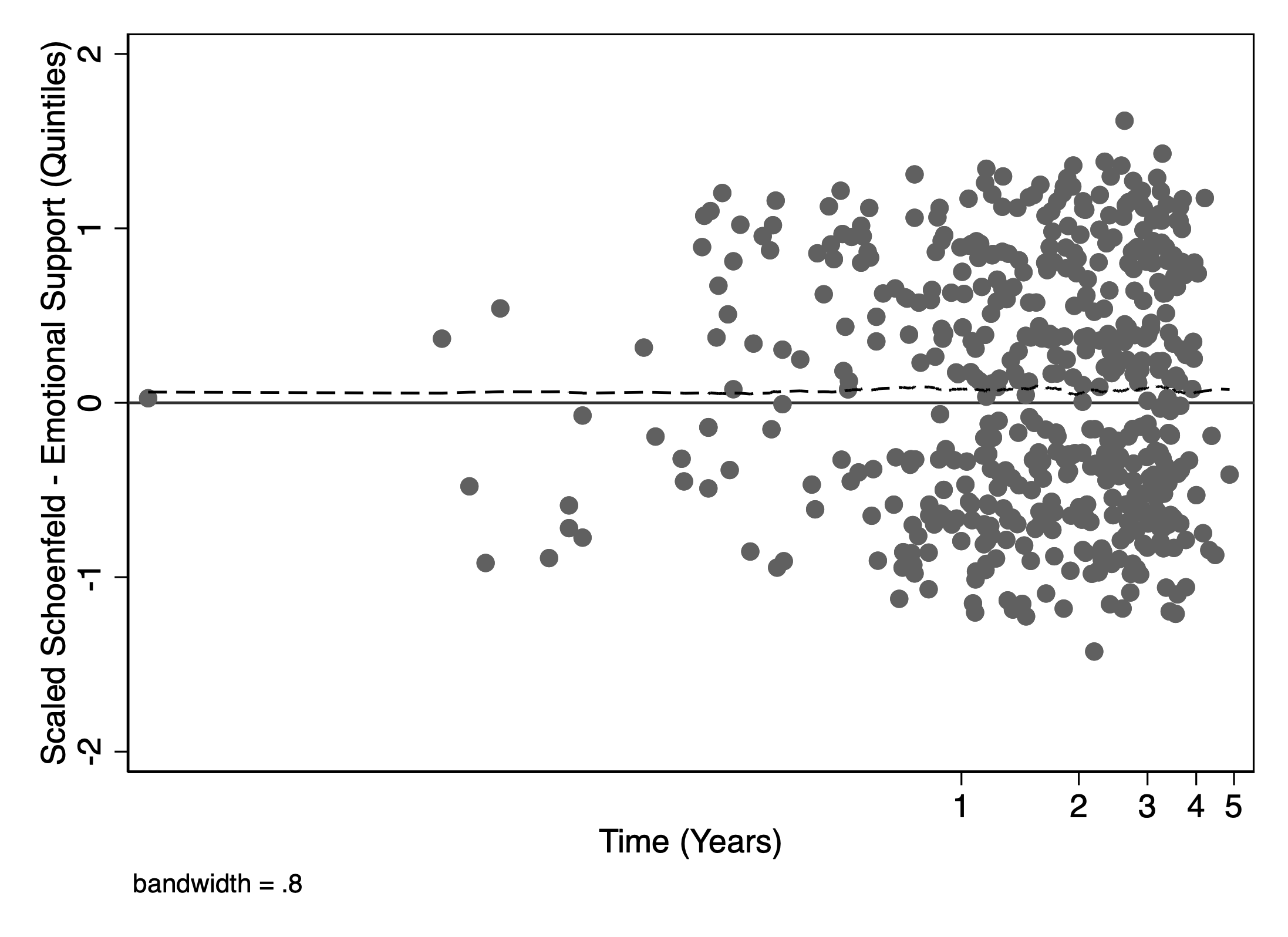

Supplement: S6 Fig — (TIF) [file pgph.0003683.s021.tif]

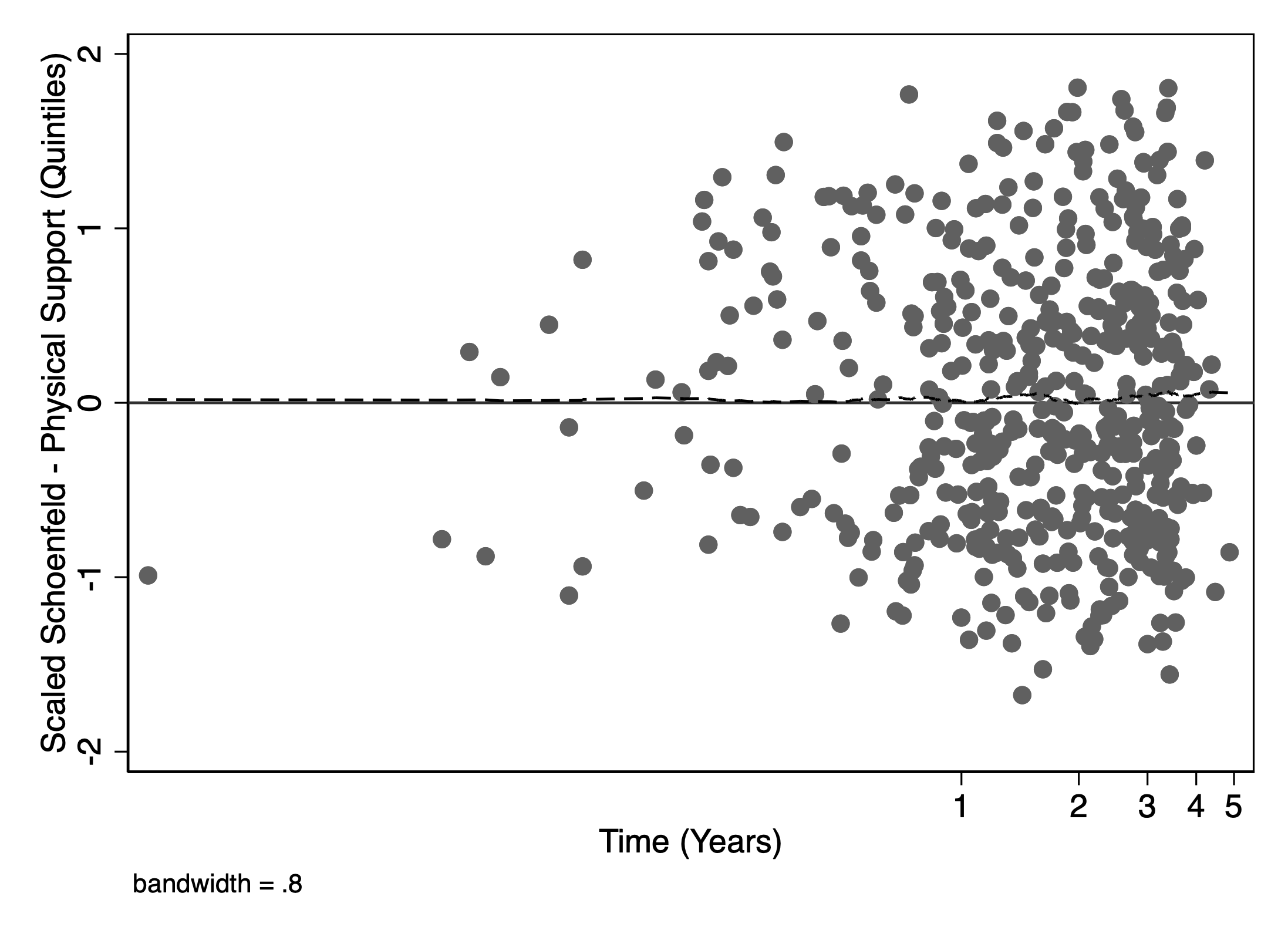

Supplement: S7 Fig — (TIF) [file pgph.0003683.s022.tif]

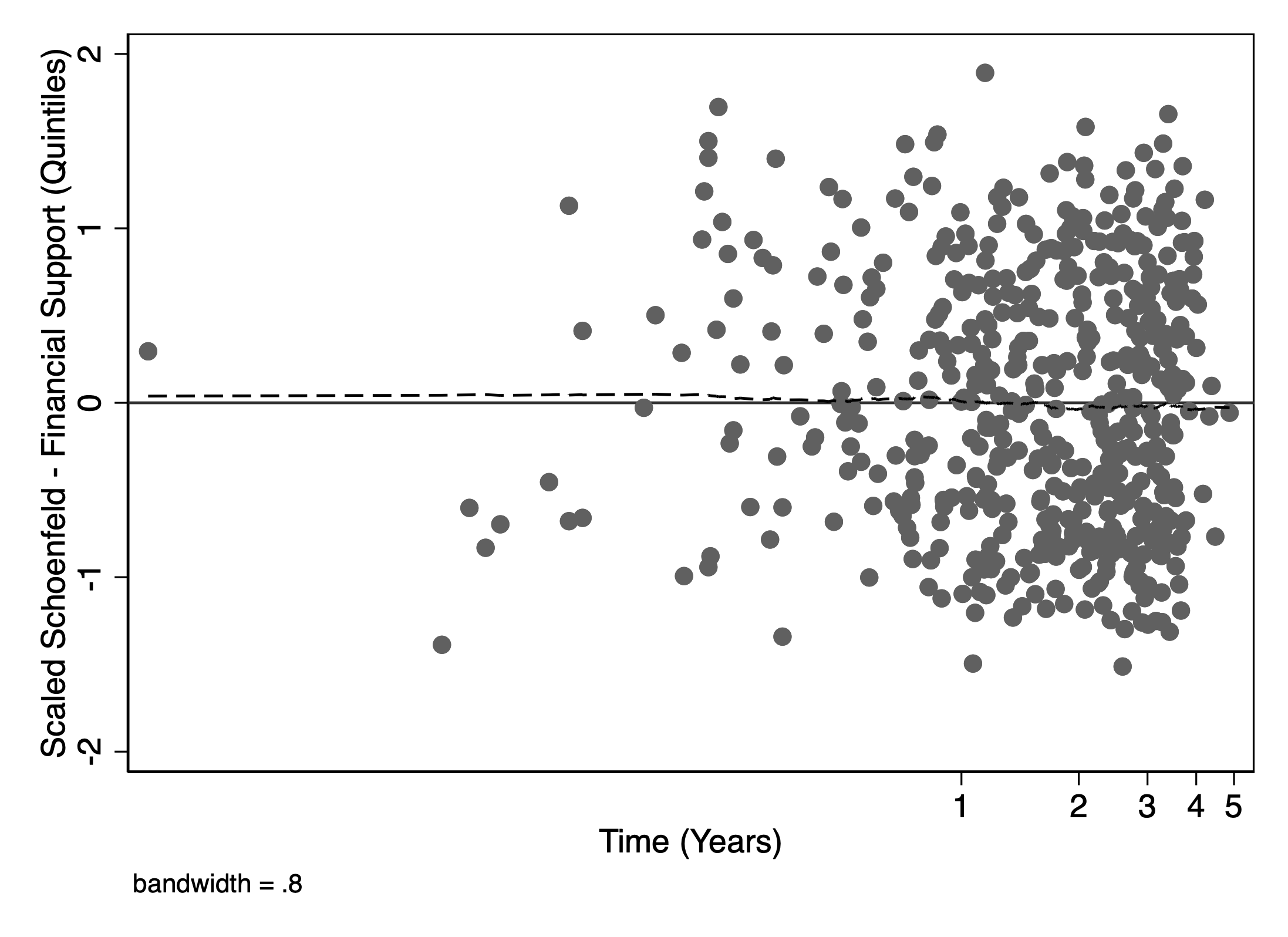

Supplement: S8 Fig — (TIF) [file pgph.0003683.s023.tif]
