# Supplementary material for: Social support receipt as a predictor of mortality: A cohort study in rural South Africa
Source: PLOS Glob Public Health. 2024 Sep 9;4(9):e0003683. doi: 10.1371/journal.pgph.0003683 (PMC11383236; doi:10.1371/journal.pgph.0003683)
Supplement: S1 Checklist — (DOCX) [file pgph.0003683.s024.docx]

Inclusivity in global research

PLOS’ policy on inclusivity in global research aims to improve transparency in the reporting of research performed outside of researchers’ own country or community and ensures that PLOS publications reporting global research adhere to high standards for research ethics and authorship. Authors of relevant research articles may be asked to complete the questionnaire below, which outlines ethical, cultural, and scientific considerations specific to inclusivity in global research. This questionnaire may be requested when researchers have travelled to a different country to conduct research, if research uses samples collected in another country, research with Indigenous populations or their lands, or if research is on cultural artefacts. Researchers travelling to another country solely to use laboratory equipment will not normally be required to complete the questionnaire. However, the questionnaire can be requested at the journal’s discretion for any submission – if you have been requested to complete this questionnaire by the PLOS journal you submitted to, please do so.

Please complete the questionnaire below and include this as a Supporting Information file with your manuscript. Note that if your paper is accepted for publication, this checklist will be published with your article in the supporting information files. Please ensure that you reference the checklist in the main body of your manuscript. We suggest adding a subsection ‘Inclusivity in global research’ to your Methods section and adding the following sentence: “Additional information regarding the ethical, cultural, and scientific considerations specific to inclusivity in global research is included in the Supporting Information (SX Checklist)”

The questions have been designed to be applicable to a wide range of study types, and there are subsections for both human subjects research and non-human subjects research. If any of the questions are not relevant to your research please mark them as “N/A” as appropriate.

**Ethical considerations, permits and authorship**

*This section is applicable to all research types.*

Provide details as to who granted permissions and/or consent for the study to take place in the Methods section of your manuscript. This should include the names of **all** ethics boards, governmental organizations, community leaders or other bodies that provided approval for the study. If individuals provided approval refer to these people by their role or title but do not list their name(s).

Reported on page number: 11

“This study received ethical approval from the Insitutional Review Board at the Harvard School of Public Health (Protocol: IRB13-1608), as well from the Human Research Ethics Committee at the University of Witwatersrand (Protocol: M141159). Informed consent was collected via verbal and written consent forms administed to all respondents prior to participation in the study.”

If there were any deviations from the study protocol after approval was obtained please provide details of these changes in the Methods section of your manuscript.

N/A – There were no deviations from the study protocol after approval was obtained.

Did this study involve local collaborators that are residents of the country where the research was conducted or members of the community studied? If you do not have any authors from said communities, please provide an explanation for this below.

Yes - The HAALSI project is a collaboration between the Harvard T.H. Chan School of Public Health in Boston, MA and the University of Witwatersrand, in Johannesburg, South Africa. In particular, collaborators at the University of Witwatersrand have been building connections with the community in the larger study area (Agincourt) since 1992 by overseeing the implementation of a socio-demographic surveillance system to improve health and well-being through rigorous longitudinal research. Their mandate is: “**In partnership with host communities and local institutions, to better understand and respond to the dynamics of health, population and social transitions in rural South and sub-Saharan Africa, in order to mount a more effective public health, public sector and social response and thereby inform national, regional and global health and development policy and practice”. In addition, three co-authors are native South Africans including** Shafika Abrahams-Gessel**,** Keletso Makofane**, and** Chodziwadziwa Whiteson Kabudula.

Everyone listed as an author should meet PLOS’ criteria for authorship and all individuals who meet these criteria should be included in the author byline, rather than the acknowledgements. For further information please see the journal’s Authorship Policy.

We have reviewed the PLOS One Authorship Policy and can confirm all co-authors meet the criteria outlined in the guidelines.

**Human subjects research (e.g. health research, medical research, cross-cultural psychology)**

Did you obtain written informed consent from a representative of the local community or region before the research took place? How did you establish who speaks for the community? Details of written informed consent obtained from study participants should be reported separately in the Methods section of your manuscript.

“The University of Witwatersrand has been working closely with its host communities for over 30 years. The Agincourt Public Engagement Office (PEO) plays a crucial role in developing constructive relationships with the community and general public, service providers, researchers, fieldworkers, data analysts, administration, and research office. The PEO is responsible for ensuring that the longitudinal community-based surveillance work rests on stable long-term relationships that need to be nurtured and fostered.

The primary relationships are between the PEO, the elected village Community Development Forums (CDFs), and the indunas of the 28 villages in the study site. The real public face of the University is its field workers, who are trained to conduct informed consent and answer questions from potential participants with sensitivity.

The PEO team is well acquainted with all CDFs, indunas, ward councillors, and local municipal officers in the field site. The team and village leadership enjoy a good working relationship. When required, the PEO meets the CDF either at regular or special meetings. Bi-annual meetings involving the CDFs, indunas, Community Advisory Board members, and Community Development Workers are held at the Agincourt offices. Meetings can be called involving the entire Traditional Council when indunas hold their joint meeting.

The PEO provides advice on community entry and runs community entry after ethics approval. It also provides community, local, district, and provincial service provider feedback. The PEO assists with dealing with ethics of practice issues arising during fieldwork and reports on community entry and feedback.”

From our collaborators website: https://www.wits.ac.za/agincourt/public-engagement/

How did members of the local community provide input on the aims of the research investigation, its methodology, and its anticipated outcome(s)?

Several PI’s are headquartered at the University of Witwatersrand and were instumental in guiding the design and content of the larger grant, as well as the overall research aims. Furthermore, several PI’s also oversee fieldwork each wave, and help direct data collection and management efforts.

When engaging with the local community, how did you ensure that the informed consent documents and other materials could be understood by local stakeholders?

We worked closely with the team at the University of Witwatersrand when designing survey questionnaires and consent forms to ensure they made sense in the larger cultural context and were capturing accurate information from all respondents. In addition, all fieldwork documents were translated and back-translated from English to Shangaan, the local language, to not only ensure the correct information was being communicated to respondents, but also that participants understood each question. Finally, individuals who live in the surrounding area, and speak both English and Shangaan, were trained and employed as fieldworkers during data collection as well. During training, fieldworkers were able to provide real-time feeback about any misunderstandings or inconsistencies between the drafted survey text and how it was actually communicated to respondents in practice.

Will the findings of the research be made available in an understandable format to stakeholders in the community where the study was conducted (e.g. via a presentation, summary report, copies of publications, etc.)? Please provide details of how this will be achieved.

Our main collaborators at the University of Witwatersrand in South Africa give presentations to the local community about research outcomes and publications. Additionally, all respondents get immediate feedback regarding point-of-care blood measures (glucose, hemoglobin, cholesterol, blood pressure, etc.) during fieldwork. Furthermore, if any measurements surpass certain thresholds, respondents are given referral letters to local hospitals or medical center to treat these conditions.

**Non-human subjects research using specimens/ animals collected as part of the study, or those housed in archival collections. Examples include archaeology, paleontology, botany and zoology.**

Did the permission you obtained from a local authority to perform the study include an agreement on access to outputs and benefit sharing? This may include procedures to enable fair distribution of the benefits and resources arising from the research performed. Please include any details of Prior Informed Consent and Benefit Sharing Agreements obtained. These may be required by field-specific regulations, for example the Convention on Biological Diversity (CBD) and the associated Nagoya Protocol.

N/A

If the material used in your study was imported, please A) provide the year it was imported and B) indicate whether permits were obtained to import/export the materials used, C) provide details of any permits obtained. If this information is not available, please indicate this.

N/A

If you used archival specimens, please state how the material used in your study was acquired by the institute it is held in and provide details of any permits obtained for the original excavations/ sample collection. If this information is not available, please indicate this.

N/A

How was the potential cultural significance of the materials collected in your study to local communities considered in your research design? Were Indigenous peoples and/or local researchers and institutions involved with archaeological excavations / collection of specimens? If so, please provide a description of their involvement.

N/A

If your manuscript includes photographs of human remains please indicate whether authors obtained permission from descendants or affiliated cultural communities to do so.

N/A
