# Supplementary material for: Social support receipt as a predictor of mortality: A cohort study in rural South Africa
Source: PLOS Glob Public Health. 2024 Sep 9;4(9):e0003683. doi: 10.1371/journal.pgph.0003683 (PMC11383236; doi:10.1371/journal.pgph.0003683)
Supplement: Attachment — Submitted filename: Response to Reviewers.docx [file pgph.0003683.s025.docx]

**Social support receipt as a predictor of mortality: a cohort study in rural South Africa**

PLOS Global Public Health – PGPH-D-23-02132. Review dated May 14th, 2024

In this document, **reviewer and editor comments** are shown in bold text, our response is in normal text with text additions underlined and italicized, and ~~removed text~~ shown with strikethrough when relevant.

**1. Does this manuscript meet PLOS Global Public Health’s** [**publication criteria**](https://journals.plos.org/globalpublichealth/s/criteria-for-publication)**? Is the manuscript technically sound, and do the data support the conclusions? The manuscript must describe methodologically and ethically rigorous research with conclusions that are appropriately drawn based on the data presented.**

**Reviewer #1: Yes**

**Reviewer #2: Yes**

**2. Has the statistical analysis been performed appropriately and rigorously?**

**Reviewer #1: Yes**

**Reviewer #2: Yes**

**3. Have the authors made all data underlying the findings in their manuscript fully available (please refer to the Data Availability Statement at the start of the manuscript PDF file)?

The** [**PLOS Data policy**](https://journals.plos.org/globalpublichealth/s/data-availability) **requires authors to make all data underlying the findings described in their manuscript fully available without restriction, with rare exception. The data should be provided as part of the manuscript or its supporting information, or deposited to a public repository. For example, in addition to summary statistics, the data points behind means, medians and variance measures should be available. If there are restrictions on publicly sharing data—e.g. participant privacy or use of data from a third party—those must be specified.**

**Reviewer #1: Yes**

**Reviewer #2: No**

HAALSI datasets are publicly available, and can be downloaded freely from either of the following data repositories:

1. ICPSR (Inter-University Consortium for Political and Social Research)
2. Harvard Dataverse

ICPSR: https://www.icpsr.umich.edu/web/NACDA/studies/36633

Harvard Dataverse: https://dataverse.harvard.edu/dataverse/haalsi

**4. Is the manuscript presented in an intelligible fashion and written in standard English?

PLOS Global Public Health does not copyedit accepted manuscripts, so the language in submitted articles must be clear, correct, and unambiguous. Any typographical or grammatical errors should be corrected at revision, so please note any specific errors here.**

**Reviewer #1: Yes**

**Reviewer #2: Yes**

**5. Review Comments to the Author

Please use the space provided to explain your answers to the questions above. You may also include additional comments for the author, including concerns about dual publication, research ethics, or publication ethics. (Please upload your review as an attachment if it exceeds 20,000 characters)**

**Reviewer #1: Dear Authors,**

**Thanks for submitting this manuscript. You tackle an important topic (social support among older people), especially in the reported context (low-income, rural, South Africa). Given the paucity of research, this article will further the knowledge and evidence base in the topic, and the recommended further research, given the unique findings is welcome. The manuscript, though discussing a complex topic with equally complex statistical tests, is also presented in an easy to understand manner, which makes it more likely to be accessed by interested stakeholders. I enjoyed reading the manuscript. Well done.**

**Reviewer #2: Social support receipt as a predictor of mortality: a cohort study in rural South Africa

I appreciated reading your important work on social support in rural South Africa. It is very timely as demographics in Africa are shifting with increased life expectancy and the rural-urban migration in search for job opportunities.

Specific comments

1. Since almost half of the sample (44.9%) had no formal education and 34.4% had some primary education. What language was used for the survey in this rural community? Could this have had an impact on responses to some of the survey questions or their understanding of what was asked? I reviewed the Cohort profile (Gómez-Olivé et al., 2018) and it only mentions that xiTsonga or English was used for obtaining informed consent but not the survey itself.**

We thank the reviewer for this important comment. The survey, in addition to the consents, were all conducted in the local language, Shangaan. Additionally, all of the respondents are Shangaan speakers. We have now clarified this in the manuscript, on page 10, where we state:

“Survey data were collected in the local language, Shangaan, via computer assisted personal interviewing (CAPI)”.

Since all of the interviews were conducted in the same language, we feel this would not impact differences in responses to questions between participants. Moreover, the survey has been piloted in each wave with items back-translated into English to ensure that respondents understand the intended meaning.

**2. Center for Epidemiological Studies-Depression (CES-D) Instrument.
I appreciate the measure of depression in this study. However, in most African cultures, the term "depression" is uncommon. How were questions 3 and 6 translated? Was there a pilot study for this CES-D instrument to check for its application in this setting?**

We appreciate this comment, and understand that depression is a concept that may not translate directly across cultures. This particular item in the CES-D 8 (“much of the time in the past week you felt depressed”) was back-translated from Shangaan to English as “most of the time last week you felt depressed.” Hence, the Shangaan language includes this word/concept in a way that allowed the back-translated for the word “depressed” to be identical to the original English. These items, along with the rest of the survey, were tested with local Shangaan speakers prior to fielding the survey.

While there was one item in the 8-item CES-D that did pose translation problems, we have handled that in the coding. This item was originally phrased as “much of the time in the past week you felt that everything you did was an effort”. However, it was back-translated as “most of the time last week you felt like you put an effort into everything that you did”. In discussions with our collaborators in Agincourt during the data analysis phase, we learned that this translation has a more positive connotation (e.g., I did everything with enthusiasm vs. everything I did was difficult) and have therefore reverse-coded this item in the analysis so that a response of “yes” is treated as not symptomatic of depression.

Additionally, while we recognize this minor issue with the CES-D translation, this item is included in our models as a covariate. It is not an ideal or perfect measure of mental health, but it is the best measure we can use to account for mental health at wave 1 and was therefore important to include as a covariate.

**3. African cultures have traditional practices where it is expected that the elders are respected and are taken care of by the younger generations even though this practice is rapidly changing with the movement to the urban areas in search of employment. Do you think this has any implications for your study?**

Thank you for this comment. We do believe the changing nature of caregiving has important implications for our analysis. Since the overall economic landscape of South Africa has forced younger individuals to travel from rural areas to urban centers for employment, this has indeed shifted some caregiving responsibilities onto older family members. Additionally, while pension plans and state grants can provide families with additional income, this extra money can create expectations that aging individuals will financially support the rest of the family, especially in resource-constrained settings like Agincourt. Although the reciprocal nature of social support may mean that elders are still taken care of by younger generations in some regard, we feel the changing nature of employment, as well as the larger economic environment, have burdened older adults with competing demands and additional care-giving responsibilities. Therefore, we hypothesized that this would lead to higher mortality for older individuals. However, given the results, we feel more work needs to be done to tease out how shifting norms around care-giving impact health and mortality for older adults. The following paragraph in the introduction touches upon many of these themes:

“Third, the country as a whole is rapidly aging with the number of individuals 65 years of age or older projected to triple by 2060 (53). This demographic and epidemiologic transition, as well as the overall economic situation in the country, have also impacted social support for aging individuals as well. Past research has found that age can have a moderating effect between social support and longevity meaning that social support likely impacts health and mortality in different ways for younger individuals compared to older individuals (54). For example, in South Africa, many older adults are left to head the household while their younger family members travel for work as a result of high unemployment rates. While this is partially due to the unique labour migration patterns in rural areas, it is also because all adults over 60 years of age become eligible for a small government pension, meaning those who receive it often become the primary source of income for the household (38,55). In turn, such grants can create expectations that individuals will be compensated with financial resources in return for supporting older family members with pensions (56–58). Although this has shifted traditions and norms around who cares for family members, it has also changed social support patterns for aging individuals by creating heightened demands for support through the pension funds, which are often the only steady cash income source for the household. (38,49,50,59,60). Overall, post-Apartheid labour and economic policies have altered the social landscape of caregiving for older individuals in many rural households which, by extension, also shifted additional support demands onto aging family members as well (61,62). Therefore, we hypothesize that greater receipt of social support will be associated with higher mortality among older individuals compared to younger respondents across all four domains due to heightened demands, distress, and care obligations to look after the rest of the household using funds from the pension scheme.”

**4. It is commendable that this study measured blood pressure and took participant's height and weight measurements for computing BMI. The overall prevalence for those who were hypertensive was 62.3%, with 66.7% of women being hypertensive versus 57% of males. In addition, 54.5% were overweight or obese. These findings, in a resource-constrained setting, are disturbing. Even though this was not the main focus of the current study, what urgent interventions would you propose to address these risk factors?**

Thank you for this comment. Indeed, the high prevalence of obesity in rural, resource-constrained settings like Agincourt is an urgent problem. In fact, a sub-project within the larger HAALSI grant studies how public policies, such as those related to reducing the amount of sugar in sweetened beverages and salt in high-sodium foods, impact population health outcomes. Our team has several papers in progress assessing such policies, including one that utilizes longitudinal urine samples to show how South African policies targeting high-salt foods has led to statistically significant reductions in sodium intake for women in the HAALSI cohort. We believe this is evidence that such policies can be useful for helping to reduce obesity and improve health outcomes in other settings as well.

**5. Since 70% were born in South Africa, were there any differences in results with those born outside of South Africa?**

Thank you for this comment. Due to its proximity to Mozambique, the wider Agincourt area has a sizeable population of Mozambican individuals who fled the country during the civil war in the 1980s. Given this history, we felt it was necessary to control for country of origin in our informational support analyses since we hypothesized the types of advice, guidance, and information individuals receive might differ by where they grew up. While there were small positive associations between being born in South Africa and mortality (HR: 1.2; 95% Confidence Interval: [0.98, 1.48]), overall, the results were statistically non-significant. Additionally, the results were virtually unchanged in all the supplementary analyses as well.

**Other comments

Lines 48-51: A long sentence. Consider revising.**

Thank you for this comment. We have split that sentence into two smaller ones to increase reader comprehension and clarity. On pages 2 & 3, the text now reads as follows:

“Possible explanations include that insufficient social support is not a strong driver of mortality risk in this setting, or that social support does not reach some necessary threshold to buffer against mortality. Additionally, it is possible that the social support measure did not capture more relevant aspects of support, or that our social support measures captured prior morbidity that attracted support before the study began.”

**Line 155: “Third, the country…”. Where are the first and second points? Consider including these for the readers to follow the arguments presented.**

Thank you for this comment. Although we had embedded the first two points in earlier paragraphs in the introduction, to improve clarity, we have edited the beginning each paragraph to start with an ordinal number to distinguish each point (“First…Second…Third…”). Additionally, we split up a long paragraph to make each of the three points to clearer to readers as well.

**Line 221: “Date and cause of death was either obtained during these calls,...” How could this have happened if someone had already passed away? Consider revising for clarity.**

Thank you for this comment. While we collect phone numbers from each respondent for future tracking, we also gather mobile numbers from other relatives, household members, and close neighbors as well. If the field team is unable to reach the actual respondent over the phone during one of the six-month check-in calls, they begin reaching out to these backup numbers. It is through these additional calls where we may find out the respondent was unable to answer the phone because they were at work, because they moved away, or if they have passed away. We have clarified this in the text on page 11, and it now reads as follows:

“Baseline respondents were tracked between waves through follow-up calls conducted every six months. In the event a respondent did not answer their phone, additional calls with family members, friends, or close neighbors were used as a back-up to track participants and their vital status. Date and cause of death was either obtained during these calls, determined separately by a verbal autopsy team, reported by a family member during wave two data collection, discovered during visits for other Agincourt studies, or taken from the HDSS.”

**Line 279: “Respondents were considered diabetic” and line 324 “were considered diabetic” consider using person-first language.**

Thank you for this comment. We have re-worded those sentences on page 14 & page 16, respectively, as follows:

“Respondents were considered as having diabetes if they self-reported ever being diagnosed, had fasting glucose levels ≥7 mmol/l (126 mg/dL), or non-fasting glucose levels ≥11.1 mmol/l (200 mg/dL)”.

“One-fifth (21%) of respondents were living with HIV, and 11% of respondents were considered as having diabetes”.

**Line 413. “Furthermore, this can subsequently can lead to increased” does not read well. Consider revising.**

Thank you for this comment. We have edited the sentence on page 17 as follows:

“Furthermore, this can subsequently ~~can~~ lead to increased dependence and further deterioration as well (91,92).”
